# A Multicohort Machine Learning Framework to Predict Mortality in Elderly Patients With Heart Disease: Insights From HARLS, SHARE, and HRS

**DOI:** 10.1155/cdr/8040700

**Published:** 2026-01-02

**Authors:** Zhiqiang Yang, Xiaohong Zhang

**Affiliations:** ^1^ Department of Cardiology, The Third Affiliated Hospital of Anhui Medical University, Hefei City, Anhui Province, China, ciss.org.cn

**Keywords:** elderly patients, heart disease, machine learning, mortality prediction, SHAP analysis

## Abstract

**Background:**

Elderly patients with heart disease face elevated mortality risk, yet predictive models specifically tailored for this population across different global regions remain limited. Current mortality prediction tools often lack cross‐cultural validation and interpretability, hindering their clinical application in diverse healthcare settings.

**Methods:**

We developed and validated machine learning models for predicting mortality in elderly heart disease patients using data from three major aging cohorts: the China Health and Retirement Longitudinal Study (CHARLS, *n* = 2130), the Survey of Health, Ageing and Retirement in Europe (SHARE, *n* = 10,928), and the Health and Retirement Study (HRS) from the United States (*n* = 4835). Boruta feature selection identified 27 common predictors across cohorts. Eleven machine learning algorithms were trained on the SHARE cohort (70% training and 30% testing) and externally validated on CHARLS and HRS cohorts. Model performance was assessed using area under the receiver operating characteristic curve (AUC), sensitivity, specificity, and calibration metrics. SHapley Additive exPlanations (SHAP) analysis was employed to interpret model predictions.

**Results:**

XGBoost demonstrated superior performance with the highest average AUC (0.798) across all datasets, showing excellent generalizability from the SHARE training set (AUC: 0.805) to internal validation (AUC: 0.799) and external validation in HRS (AUC: 0.821) and CHARLS (AUC: 0.770) cohorts. Age consistently emerged as the most influential predictor across all cohorts (SHAP values: 0.056–0.102), followed by gender, moderate physical activity, and self‐rated health, though their relative importance varied by cohort. Feature dependence analysis revealed important nonlinear relationships, including U‐shaped associations between grip strength and mortality risk.

**Conclusion:**

Our multicohort machine learning approach successfully developed a robust, interpretable model for predicting mortality in elderly heart disease patients across diverse global populations. The model′s strong performance in external validation demonstrates its potential for cross‐cultural clinical application, while SHAP analysis provides valuable insights into population‐specific risk factors that could guide targeted interventions.

## 1. Introduction

Cardiovascular disease (CVD) remains the leading cause of morbidity and mortality globally, disproportionately affecting older adults with preexisting cardiac conditions. As population aging accelerates worldwide, the burden of managing heart disease in older individuals has become a pressing public health concern. In the United States alone, it accounts for the highest burden of disease and death. According to the 2017–2020 National Health and Nutrition Examination Survey (NHANES), approximately 48.6% of US adults aged ≥ 20 years are affected by at least one form of CVD, including coronary heart disease, heart failure, stroke, or hypertension. Even excluding hypertension, the burden remains substantial. CVD causes more deaths than cancer among individuals under 85 years (596,786 vs. 502,847 deaths), underscoring its major contribution to premature mortality. Furthermore, life expectancy in the US declined sharply from 78.8 years in 2019 to 76.1 years in 2021—a 2.7‐year reduction—partially driven by cardiovascular conditions [1]. While these figures highlight the urgent cardiovascular burden in the United States, similar or even greater challenges exist worldwide. As the global population ages, heart disease is becoming an increasingly critical health challenge. By 2025, the number of adults aged ≥ 60 is projected to double, and by 2050, nearly half of the world′s CVD burden is expected to concentrate in the Asia–Pacific region [2–4]. In China, the situation is equally concerning. According to official reports, CVD ranks first in both incidence and mortality among urban and rural residents, surpassing cancer and other major diseases [5]. These statistics underscore the global imperative to develop accurate, population‐specific tools for mortality prediction, particularly to identify high‐risk individuals and guide timely intervention among older adults with established heart disease.

In elderly patients, prognosis is often shaped by a complex interplay of demographic, clinical, behavioral, functional, and psychosocial factors. Accurate prediction of mortality risk in this population is essential for guiding clinical decision‐making, allocating healthcare resources, and designing personalized interventions. However, existing risk prediction tools—such as the Framingham Risk Score and GRACE score—were primarily developed in middle‐aged populations and have limited applicability among older adults, particularly those with multimorbidity or functional impairment [6, 7].

Recent advances in machine learning (ML) have opened new opportunities for developing predictive models capable of capturing complex, nonlinear relationships among diverse clinical features [8]. ML‐based models have shown promise in improving risk stratification across various cardiovascular conditions, including heart failure, atrial fibrillation, and myocardial infarction [9–13]. Nevertheless, most existing models have been derived from single‐country datasets and are rarely validated in diverse geographic or ethnic populations [14]. Furthermore, many ML models operate as “black boxes,” limiting their clinical utility due to a lack of transparency and interpretability—especially when applied to heterogeneous and vulnerable populations like the elderly [15].

In the context of global aging, there is a critical need for robust, generalizable, and interpretable predictive models that can perform reliably across different sociocultural environments and healthcare systems. Multicohort, multinational studies offer a unique opportunity to develop such tools. However, cross‐cohort harmonization of variables, differences in measurement instruments, and inconsistent definitions of clinical outcomes pose substantial analytical challenges. Moreover, few studies to date have systematically examined whether ML models trained in one population can accurately predict outcomes in another, particularly in older adults with heart disease.

To address these limitations, we developed and validated a multicohort ML framework for predicting all‐cause mortality in elderly individuals with heart disease, using harmonized data from three nationally representative aging cohorts: the China Health and Retirement Longitudinal Study (CHARLS); the Survey of Health, Ageing and Retirement in Europe (SHARE); and the Health and Retirement Study (HRS) from the United States [16–18]. These datasets encompass elderly individuals with baseline heart disease and longitudinal follow‐up on mortality outcomes.

We applied a rigorous and scalable ML pipeline that included (1) variable harmonization across cohorts, (2) feature selection using Boruta, (3) model development and internal validation using the SHARE dataset, and (4) external validation in the CHARLS and HRS datasets. To ensure transparency and clinical relevance, we applied SHapley Additive exPlanations (SHAP) analysis to interpret global and local feature contributions across populations. By leveraging the complementary strengths of three longitudinal cohort studies and integrating explainable AI methods, our study provides novel insights into common and population‐specific drivers of mortality risk in elderly patients with heart disease.

This work is aimed at informing the development of globally applicable, interpretable prediction models that can facilitate clinical risk assessment in older adults and support data‐driven, individualized care strategies in cardiogeriatrics. To our knowledge, this is the first study to develop and externally validate a SHAP‐interpretable ML model for mortality prediction in elderly heart disease patients using harmonized data from three large‐scale cohorts spanning Asia, Europe, and North America, demonstrating strong potential for global clinical implementation.

## 2. Methods

### 2.1. Data Sources and Study Population

#### 2.1.1. Data Sources

This study utilized data from three major longitudinal studies on aging: the CHARLS, the HRS from the United States, and the SHARE.

The CHARLS is a nationally representative longitudinal survey that aims to collect high‐quality microdata on Chinese adults aged 45 and older to analyze aging‐related issues in China. The national baseline survey was conducted in 2011, covering 150 county‐level units, 450 village‐level units, and approximately 17,000 individuals from 10,000 households. Follow‐up surveys were conducted in 2013, 2015, 2018, 2020, and 2021–2023. This study utilized data from Wave 1 (2012) through Wave 5 (2020), with the latest data update released in November 2023 [16, 19]. All CHARLS survey protocols were approved by the Biomedical Ethics Committee of Peking University (Approval Number: IRB00001052‐11015).

The HRS is a nationally representative longitudinal survey of Americans aged 50 and older, initiated in 1992. The survey is conducted biennially and tracks approximately 20,000 participants, providing detailed information on health status, cognitive function, employment history, income, assets, insurance, and family structure. This study used data from Wave 10 (2010) through Wave 15 (2020), with the latest publicly available data from the 2020 survey released in September 2022 [17]. The HRS protocol was approved by the Institutional Review Board at the University of Michigan (Approval Number: HUM00061128).

The SHARE is a cross‐national longitudinal study that collects data on health, socioeconomic status, and social networks of Europeans aged 50 and older. Initiated in 2004, SHARE covers 28 European countries and approximately 140,000 participants, with biennial data collection. This study utilized data from Wave 5 (2013) through Wave 8 (2020), with the latest publicly available data from the 2020 survey (including a special COVID‐19 survey) released in December 2022 [18]. SHARE was approved by the Ethics Committee of the University of Munich (Approval Number: 142/16_S).

#### 2.1.2. Study Population and Baseline Characteristics

We identified individuals who had been diagnosed with heart disease at baseline in each dataset: Wave 1 (2012) for CHARLS, Wave 5 (2013) for SHARE, and Wave 10 (2010) for HRS. Heart disease diagnosis was determined based on self‐reported physician diagnosis. Baseline demographic and clinical characteristics were collected, including age, gender, marital status, education level, comorbid conditions (hypertension, diabetes, cancer, lung disease, stroke, and arthritis), health behaviors (smoking, alcohol consumption, and physical activity), physical measurements (height, weight, BMI, and grip strength), self‐rated health, cognitive function, depression symptoms, and measures of functional status. The survival status of participants was determined through follow‐up surveys up to the latest available wave in each dataset. Survival time was calculated in months from baseline to either the date of death or the end of follow‐up. Participants who remained alive at the end of follow‐up were censored.

#### 2.1.3. Data Preprocessing and Harmonization

The complete list of variables can be found in Table 1. All variables across the three cohorts were strictly coordinated (Table 1 displays unified variable names and classifications). Highly correlated variables were identified and addressed through VIF analysis to ensure the validity of model inputs. For example, total recall was identified as a linear combination of immediate recall and delayed recall, and it was excluded from the final model.

**Table 1:** Baseline characteristics of heart disease patients by database and survival status.

		**CHARLS**			**HRS**			**SHARE**		
**Characteristics**	**Level**	**Survived (** **n** = 1711**)**	**Death (** **n** = 419**)**	**p** **value**	**Survived (** **n** = 2407**)**	**Death (** **n** = 2428**)**	**p** **value**	**Survived (** **n** = 7745**)**	**Death (** **n** = 3183**)**	**p** **value**
Age (years)		60.00 [54.00, 66.00]	70.00 [63.00, 76.00]	< 0.001	67.00 [58.00, 74.00]	78.00 [72.00, 85.00]	< 0.001	70.00 [63.00, 77.00]	79.00 [73.00, 84.00]	< 0.001

Gender	Female	1066 (62.3)	203 (48.4)	< 0.001	1246 (51.8)	1224 (50.4)	0.361	3848 (49.7)	1377 (43.3)	< 0.001
	Male	645 (37.7)	216 (51.6)		1161 (48.2)	1204 (49.6)		3897 (50.3)	1806 (56.7)	

Hypertension	No	840 (49.1)	177 (42.2)	0.014	720 (29.9)	459 (18.9)	< 0.001	2717 (35.1)	930 (29.2)	< 0.001
	Yes	871 (50.9)	242 (57.8)		1687 (70.1)	1969 (81.1)		5028 (64.9)	2253 (70.8)	

Diabetes	No	1494 (87.3)	343 (81.9)	0.005	1739 (72.2)	1511 (62.2)	< 0.001	5898 (76.2)	2171 (68.2)	< 0.001
	Yes	217 (12.7)	76 (18.1)		668 (27.8)	917 (37.8)		1847 (23.8)	1012 (31.8)	

Stroke	No	1627 (95.1)	388 (92.6)	0.057	2140 (88.9)	1799 (74.1)	< 0.001	6741 (87.0)	2477 (77.8)	< 0.001
	Yes	84 (4.9)	31 (7.4)		267 (11.1)	629 (25.9)		1004 (13.0)	706 (22.2)	

Cancer	No	1685 (98.5)	414 (98.8)	0.785	2061 (85.6)	1834 (75.5)	< 0.001	6850 (88.4)	2668 (83.8)	< 0.001
	Yes	26 (1.5)	5 (1.2)		346 (14.4)	594 (24.5)		895 (11.6)	515 (16.2)	

Lung disease	No	1459 (85.3)	301 (71.8)	< 0.001	2125 (88.3)	1883 (77.6)	< 0.001	6593 (85.1)	2471 (77.6)	< 0.001
	Yes	252 (14.7)	118 (28.2)		282 (11.7)	545 (22.4)		1152 (14.9)	712 (22.4)	

Arthritis	No	889 (52.0)	247 (58.9)	0.012	858 (35.6)	568 (23.4)	< 0.001	4401 (56.8)	1645 (51.7)	< 0.001
	Yes	822 (48.0)	172 (41.1)		1549 (64.4)	1860 (76.6)		3344 (43.2)	1538 (48.3)	

Hypertension medication	No	1037 (60.6)	231 (55.1)	0.046	783 (32.5)	537 (22.1)	< 0.001	2911 (37.6)	1078 (33.9)	< 0.001
	Yes	674 (39.4)	188 (44.9)		1624 (67.5)	1891 (77.9)		4834 (62.4)	2105 (66.1)	

Diabetes medication	No	1585 (92.6)	360 (85.9)	< 0.001	1843 (76.6)	1620 (66.7)	< 0.001	6253 (80.7)	2412 (75.8)	< 0.001
	Yes	126 (7.4)	59 (14.1)		564 (23.4)	808 (33.3)		1492 (19.3)	771 (24.2)	

Depression	No	888 (51.9)	200 (47.7)	0.140	1950 (81.0)	1856 (76.4)	< 0.001	5025 (64.9)	1667 (52.4)	< 0.001
	Yes	823 (48.1)	219 (52.3)		457 (19.0)	572 (23.6)		2720 (35.1)	1516 (47.6)	

Dressing difficulty	No	1602 (93.6)	341 (81.4)	< 0.001	2092 (86.9)	1764 (72.7)	< 0.001	6788 (87.6)	2278 (71.6)	< 0.001
	Yes	109 (6.4)	78 (18.6)		315 (13.1)	664 (27.3)		957 (12.4)	905 (28.4)	

Bathing difficulty	No	1565 (91.5)	313 (74.7)	< 0.001	2228 (92.6)	1812 (74.6)	< 0.001	7030 (90.8)	2251 (70.7)	< 0.001
	Yes	146 (8.5)	106 (25.3)		179 (7.4)	616 (25.4)		715 (9.2)	932 (29.3)	

Eating difficulty	No	1667 (97.4)	375 (89.5)	< 0.001	2329 (96.8)	2094 (86.2)	< 0.001	7526 (97.2)	2833 (89.0)	< 0.001
	Yes	44 (2.6)	44 (10.5)		78 (3.2)	334 (13.8)		219 (2.8)	350 (11.0)	

Bed difficulty	No	1594 (93.2)	345 (82.3)	< 0.001	2190 (91.0)	2000 (82.4)	< 0.001	7276 (93.9)	2637 (82.8)	< 0.001
	Yes	117 (6.8)	74 (17.7)		217 (9.0)	428 (17.6)		469 (6.1)	546 (17.2)	

Toilet difficulty	No	1421 (83.1)	297 (70.9)	< 0.001	2224 (92.4)	1973 (81.3)	< 0.001	7467 (96.4)	2753 (86.5)	< 0.001
	Yes	290 (16.9)	122 (29.1)		183 (7.6)	455 (18.7)		278 (3.6)	430 (13.5)	

Immediate recall score		4.00 [3.00, 5.00]	4.00 [3.00, 5.00]	< 0.001	5.00 [4.00, 6.00]	4.00 [3.00, 5.00]	< 0.001	5.00 [4.00, 6.00]	4.00 [3.00, 5.00]	< 0.001

Delayed recall score		3.00 [2.00, 4.00]	3.00 [1.00, 4.00]	< 0.001	4.00 [3.00, 5.00]	3.00 [2.00, 4.00]	< 0.001	4.00 [2.00, 5.00]	2.00 [0.00, 4.00]	< 0.001

Total recall score		7.00 [5.00, 9.00]	6.00 [4.00, 8.00]	< 0.001	10.00 [8.00, 12.00]	8.00 [6.00, 10.00]	< 0.001	9.00 [6.00, 11.00]	6.00 [4.00, 9.00]	< 0.001

Serial subtraction score	0	85 (5.0)	36 (8.6)	< 0.001	182 (7.6)	264 (10.9)	< 0.001	155 (2.0)	127 (4.0)	< 0.001
	1	252 (14.7)	77 (18.4)		254 (10.6)	308 (12.7)		444 (5.7)	326 (10.2)	
	2	134 (7.8)	74 (17.7)		205 (8.5)	297 (12.2)		419 (5.4)	319 (10.0)	
	3	219 (12.8)	60 (14.3)		323 (13.4)	423 (17.4)		814 (10.5)	462 (14.5)	
	4	300 (17.5)	66 (15.8)		498 (20.7)	480 (19.8)		1274 (16.4)	570 (17.9)	
	5	721 (42.1)	106 (25.3)		945 (39.3)	656 (27.0)		4639 (59.9)	1379 (43.3)	

Orientation score	1	156 (9.1)	53 (12.6)	< 0.001	13 (0.5)	56 (2.3)	< 0.001	45 (0.6)	50 (1.6)	< 0.001
	2	237 (13.9)	88 (21.0)		58 (2.4)	162 (6.7)		165 (2.1)	223 (7.0)	
	3	465 (27.2)	140 (33.4)		447 (18.6)	674 (27.8)		999 (12.9)	831 (26.1)	
	4	853 (49.9)	138 (32.9)		1889 (78.5)	1536 (63.3)		6536 (84.4)	2079 (65.3)	

Ever smoked	No	1126 (65.8)	227 (54.2)	< 0.001	971 (40.3)	861 (35.5)	0.001	4045 (52.2)	1638 (51.5)	0.479
	Yes	585 (34.2)	192 (45.8)		1436 (59.7)	1567 (64.5)		3700 (47.8)	1545 (48.5)	

Current smoking	No	1323 (77.3)	312 (74.5)	0.239	2076 (86.2)	2155 (88.8)	0.010	6688 (86.4)	2768 (87.0)	0.414
	Yes	388 (22.7)	107 (25.5)		331 (13.8)	273 (11.2)		1057 (13.6)	415 (13.0)	

Alcohol consumption	No	1311 (76.6)	326 (77.8)	0.653	1090 (45.3)	1518 (62.5)	< 0.001	4697 (60.6)	2197 (69.0)	< 0.001
	Yes	400 (23.4)	93 (22.2)		1317 (54.7)	910 (37.5)		3048 (39.4)	986 (31.0)	

Height (m)		1.58 [1.53, 1.65]	1.59 [1.52, 1.65]	0.894	1.66 [1.59, 1.74]	1.65 [1.57, 1.72]	< 0.001	1.68 [1.61, 1.75]	1.68 [1.60, 1.74]	< 0.001
Weight (kg)		62.50 [54.50, 70.62]	59.56 [51.50, 67.57]	< 0.001	81.65 [69.63, 95.25]	73.37 [64.37, 86.40]	< 0.001	78.00 [69.00, 89.00]	75.00 [65.00, 85.00]	< 0.001

BMI (kg/m^2^)		24.69 [22.27, 27.52]	23.55 [21.09, 26.07]	< 0.001	29.21 [25.91, 33.68]	27.11 [24.68, 30.88]	< 0.001	27.31 [24.62, 30.47]	26.45 [23.88, 29.76]	< 0.001

Grip strength (kg)		29.50 [23.00, 37.00]	25.00 [18.00, 32.00]	< 0.001	30.50 [23.00, 39.55]	23.50 [17.50, 31.50]	< 0.001	27.00 [20.00, 38.00]	23.00 [15.90, 31.00]	< 0.001

Currently working	No	897 (52.4)	310 (74.0)	< 0.001	1557 (64.7)	2225 (91.6)	< 0.001	6258 (80.8)	3012 (94.6)	< 0.001
	Yes	814 (47.6)	109 (26.0)		850 (35.3)	203 (8.4)		1487 (19.2)	171 (5.4)	

#### 2.1.4. Data Cleaning and Missing Value Handling

Data preprocessing was performed using R Version 4.4.2. Variables with more than 30% missing values were excluded from the analysis. For the remaining variables, missing values were imputed using multiple imputation with the mice package (Version 3.14.0). The high complete case rate indicates that the impact of loss to follow‐up is limited. For variables with missing data, we used five imputations to ensure all patients were included in the analysis, thereby minimizing loss to follow‐up bias. Distributional checks confirmed that the imputed data maintained consistency with the original variable distributions. We employed different imputation methods based on the variable type: predictive mean matching (PMM) was applied for continuous variables, logistic regression for binary variables, and multinomial logistic regression for categorical variables with more than two categories. Five imputed datasets were created, and the final imputed values were derived by averaging across these datasets for continuous variables and using the mode for categorical variables. Outliers were identified using the three‐sigma rule and treated as missing values before imputation. After imputation, we verified that integer variables maintained their integer properties and that the imputed values were within plausible ranges for each variable.

#### 2.1.5. Data Harmonization Across Cohorts

To enable cross‐cohort comparison and analysis, variables from the three datasets were harmonized to ensure consistency in naming, coding, and measurement. Key variables were standardized across cohorts, including demographic characteristics (age, gender, marital status, and education), health conditions (heart disease, hypertension, diabetes, cancer, lung disease, stroke, and arthritis), health behaviors (smoking, alcohol consumption, and physical activity), physical measurements (height, weight, BMI, and grip strength), cognitive function, depression measures, and functional status indicators. Binary health conditions were coded as “Yes” or “No” across all datasets.

### 2.2. Feature Selection

Feature selection was performed using the Boruta algorithm, an all‐relevant feature selection approach based on iteratively comparing original and shadow features using random forests. Boruta determines feature importance by comparing the importance of original attributes with those of randomly permuted copies (shadow features) [20]. Variables that consistently show higher importance than the shadow features are deemed important. Given that our data has some missing values (as shown in “1_Variable missing rate”: The average missing rate is shown as 2.38%), Boruta enhances the stability of feature selection through multiple iterations and randomness.

For each dataset, we ran Boruta with 500 iterations and used survival status as the target variable. Features were classified into three categories: confirmed important, tentatively important, and rejected. Features with a mean importance *Z*‐score significantly higher than the highest shadow feature were confirmed as important, while those with a mean importance *Z*‐score higher than but not significantly different from the highest shadow feature were considered tentatively important. The common set of features confirmed as important across all three cohorts was used for subsequent model development.

### 2.3. ML Model Development

#### 2.3.1. Model Training and Validation

Taking all factors into account, the SHARE cohort has advantages in terms of sample size, data quality, and population representativeness. Therefore, we used the SHARE cohort as the primary dataset for model development and internal validation, with a 70:30 split for training and testing. The CHARLS and HRS cohorts were used for external validation. A comprehensive ML pipeline was implemented using the caret package in R.

First, run Boruta on three independent datasets, respectively. Then, select the common important features, and the 70:30 division of SHARE is carried out. Use 70% training extreme gradient boosting (XGBoost) and 30% internal validation. Last, CHARLS and HRS are used as completely independent external validations. Feature selection is completed before data segmentation and uses common features across datasets, so there is no risk of data leakage.

To address potential class imbalance in the survival outcome, we evaluated the class ratio (deaths/alive) in the SHARE dataset. We implemented a robust cross‐validation strategy using 10‐fold cross‐validation with five repetitions to ensure reliable model evaluation and selection.

We trained and evaluated 11 different ML models: random forest, the XGBoost, support vector machine (SVM) with radial kernel, neural network, gradient boosting machine (GBM), naive Bayes, *k*‐nearest neighbors (KNNs), classification and regression trees (CARTs), the adaptive boosting (AdaBoost), C5.0 decision trees, and conditional inference trees.

To prevent overfitting, we employed L2 regularization and grid‐based hyperparameter tuning for the following models: random forest, SVM, KNN, C5.0, neural network, XGBoost, and ranger. For random forest, we limited the number of trees to 50, increased the minimum node size to 100, restricted the maximum number of nodes to 8, and used sampling without replacement to enforce stronger regularization. For XGBoost, we tuned the learning rate (eta), maximum tree depth, and number of boosting rounds. Due to the collinearity of the data, XGBoost automatically selects the most informative variables through the tree splitting mechanism. Therefore, the XGBoost model can effectively handle collinear variables, ensuring that the model still maintains good predictive performance. For SVM, we optimized the sigma and cost parameters. For the neural network, we tuned the number of hidden units and weight decay. The hyperparameter tuning process was guided by the goal of minimizing the difference in area under the ROC curve (AUC) between the training and testing sets, ensuring this difference remained below 5% to avoid both overfitting and underfitting.

#### 2.3.2. Model Evaluation

Models were evaluated using several performance metrics: AUC, sensitivity, specificity, and accuracy. Moreover, we compared XGBoost with two widely used scoring systems (Charlson Index and Framingham Risk Score). We calculated these metrics for the SHARE training set, SHARE testing set, and both external validation cohorts (CHARLS and HRS). For each model, we identified the optimal threshold that maximized the Youden index (sensitivity + specificity − 1).

ROC curves were plotted for all models across all datasets, with models ordered by their average AUC across all datasets. A heat map was created to visualize AUC values for each model across datasets, facilitating direct comparison of model performance and generalizability. The best performing model was selected based on the highest average AUC across all datasets, with particular emphasis on external validation performance.

#### 2.3.3. Model Interpretability

We employed SHAP to interpret the best performing model using the kernelshap and shapviz packages in R. SHAP values quantify the contribution of each feature to the prediction for each individual instance, allowing both global and local interpretations of the model.

### 2.4. Global Interpretability

For global interpretation, we computed mean absolute SHAP values for each feature across all instances in each dataset, providing a measure of overall feature importance. Features were ranked by their mean absolute SHAP values, and the results were visualized using bar plots.

SHAP summary plots (beeswarm plots) were created to show the distribution of SHAP values for each feature across all instances, with color coding to indicate the feature value. These plots reveal not only the importance of each feature but also how different feature values impact the prediction.

### 2.5. Local Interpretability

For local interpretation, we created SHAP force plots to visualize how each feature contributes to the prediction for each individual instance. We also generated SHAP dependence plots to explore the relationship between feature values and their SHAP values, revealing how the model′s predictions change as a function of input features.

SHAP waterfall plots were created for representative high‐risk and low‐risk patients from each dataset, showing how each feature contributes to pushing the prediction higher or lower than the base value.

### 2.6. Statistical Analysis

Statistical analyses were performed using R Version 4.4.2. Continuous variables were presented as median and interquartile range [Q1, Q3], and categorical variables were presented as frequencies and percentages.

For ML model evaluation, we used the AUC as the primary performance metric, with 95% confidence intervals calculated using the bootstrap method (1000 replications). Additional evaluation metrics included sensitivity, specificity, accuracy, and precision. All *p* value adjustments were performed using the Benjamini–Hochberg procedure to ensure appropriate control of the false discovery rate.

## 3. Results

### 3.1. Study Population

Ultimately, we included 17,893 samples, with 12,387 complete data cases (2130, 4835, and 10,928 samples for CHARLS, HRS, and SHARE datasets, respectively). The samples of the training set and validation set in the SHARE dataset are 7651 (2229 deaths) and 3277 (954 deaths). The median follow‐up times were 109, 113, and 90 months for CHARLS, HRS, and SHARE datasets, respectively. The narrower IQR, especially CHARLS with 4 months, indicates that most patients completed full follow‐up, with a low loss‐to‐follow‐up rate. All three cohorts recorded clear mortality outcomes (Table 1). These internationally recognized longitudinal cohort studies all employed standardized death confirmation procedures, including regular linkage to national death registries, family interviews, and medical record verification.

We provided a detailed report on missing data. There is bias caused by missing data: selective missingness: Missing data for cognitive tests and physical function measurements may be related to health status, as frail individuals may be unable to complete the tests. Information bias: The mortality rate for complete cases (24.63%) is lower than that for imputed cases (33.70%), suggesting that healthier individuals tend to have more complete data. Mitigation measures: We used five imputation iterations and PMM to preserve the distribution characteristics of the variables. These analyses indicate that, although challenges such as class imbalance and missing data exist, our approach effectively addresses these issues through model selection, large sample size, and appropriate imputation strategies. The model′s robustness was confirmed through external validation.

Our data does exhibit varying degrees of class imbalance. Calibration analysis revealed the impact of imbalance. XGBoost automatically handles class imbalance through the scale_pos_weight parameter, eliminating the need for external balancing techniques. The SHARE training set contains 7651 samples and 2229 events, providing enough data for the model to learn. Also, manual balancing could result in poor performance on real‐world data. Our model still achieved an AUC of 0.7695 in the imbalanced CHARLS dataset (with a 19.67% event rate) (Table 2). This is the reason why data balancing technology was not used.

**Table 2:** Comparison of performance metrics between different models.

**Dataset**	**SHARE_Test**	**CHARLS**	**HRS**
*N*	3277	2130	4835
Events	954	419	2428
Event_Rate	0.291	0.197	0.502
XGBoost_AUC	0.7988	0.7695	0.8211
Charlson_AUC	0.7275	0.7509	0.7804
Framingham_AUC	0.6631	0.7261	0.7227
XGB_vs_Charlson_P	0	0.075	0
XGB_vs_Framingham_P	0	0	0
NRI_vs_Charlson	0.1899	0.3351	0.3292
IDI_vs_Charlson	0.2091	0.1187	0.1752

**Figure 1:**
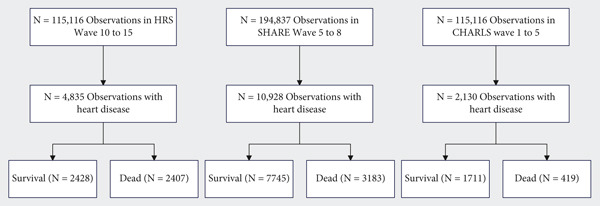
Study population flow diagram. This figure illustrates the study population selection process for each of the three databases (CHARLS, SHARE, and HRS). It details the initial sample sizes, inclusion criteria, exclusion criteria, and final analysis cohorts. Abbreviations: HRS, the Health and Retirement Study; SHARE, the Survey of Health, Ageing and Retirement in Europe; CHARLS, the China Health and Retirement Longitudinal Study.

The flow of study participants is illustrated in Figure 1. From the initial observations in each database (115,116 in HRS Wave 10–15, 194,837 in SHARE Wave 5–8, and 115,116 in CHARLS Wave 1–5), we identified patients with heart disease at baseline: 4835 from HRS, 10,928 from SHARE, and 2130 from CHARLS. After follow‐up, mortality outcomes were documented with 2407 deaths (49.8%) in HRS, 3183 deaths (29.1%) in SHARE, and 419 deaths (19.7%) in CHARLS. For details on the results of category balance and missing data, please refer to the supporting information.

The baseline characteristics of heart disease patients from the three databases are presented in Table 3. Significant differences were observed in demographic characteristics across the databases. Patients from CHARLS were notably younger (median age 61.0 years) compared to HRS (73.0 years) and SHARE (73.0 years) (*p* < 0.001). And gender composition varied significantly, with CHARLS having the highest proportion of females (59.6%), followed by HRS (51.1%) and SHARE (47.8%) (*p* < 0.001). Educational attainment also differed substantially, with 86.2% of CHARLS participants having primary education or below, compared to 49.2% in SHARE and only 23.1% in HRS (*p* < 0.001).

**Table 3:** Comparison of baseline characteristics between different databases.

**Characteristics**	**Level**	**Overall (** **n** = 17893**)**	**CHARLS (** **n** = 2130**)**	**HRS (** **n** = 4835**)**	**SHARE (** **n** = 10928**)**	**p** **value**	**SMD**
Age (years) (median [Q1, Q3])		72.0 [63.0, 79.0]	61.0 [55.0, 69.0]	73.0 [63.0, 80.0]	73.0 [65.0, 80.0]	< 0.001	0.7

Gender	Female	8964 (50.1)	1269 (59.6)	2470 (51.1)	5225 (47.8)	< 0.001	0.2
	Male	8929 (49.9)	861 (40.4)	2365 (48.9)	5703 (52.2)		

Education level	Primary education or below	8333 (46.6)	1837 (86.2)	1116 (23.1)	5380 (49.2)	< 0.001	1.0
	Secondary education	6734 (37.6)	230 (10.8)	2867 (59.3)	3637 (33.3)		
	Tertiary education	2826 (15.8)	63 (3.0)	852 (17.6)	1911 (17.5)		

BMI (kg/m^2^) (median [Q1, Q3])		27.0 [24.2, 30.5]	24.5 [22.0, 27.3]	28.2 [25.2, 32.4]	27.1 [24.4, 30.4]	< 0.001	0.6

Height (m) (median [Q1, Q3])		1.7 [1.6, 1.7]	1.6 [1.5, 1.7]	1.7 [1.6, 1.7]	1.7 [1.6, 1.8]	< 0.001	0.7

Weight (kg) (median [Q1, Q3])		75.0 [65.0, 86.9]	62.0 [54.0, 70.1]	77.6 [66.4, 91.2]	77.0 [68.0, 87.0]	< 0.001	0.8

Grip strength (kg) (median [Q1, Q3])		26.5 [19.3, 35.9]	28.5 [22.0, 36.0]	27.0 [20.0, 35.5]	26.0 [18.8, 36.0]	< 0.001	0.1

Stroke	No	15,172 (84.8)	2015 (94.6)	3939 (81.5)	9218 (84.4)	< 0.001	0.3
	Yes	2721 (15.2)	115 (5.4)	896 (18.5)	1710 (15.6)		

Hypertension	No	5843 (32.7)	1017 (47.7)	1179 (24.4)	3647 (33.4)	< 0.001	0.3
	Yes	12,050 (67.3)	1113 (52.3)	3656 (75.6)	7281 (66.6)		

Diabetes	No	13,156 (73.5)	1837 (86.2)	3250 (67.2)	8069 (73.8)	< 0.001	0.3
	Yes	4737 (26.5)	293 (13.8)	1585 (32.8)	2859 (26.2)		

Cancer	No	15,512 (86.7)	2099 (98.5)	3895 (80.6)	9518 (87.1)	< 0.001	0.4
	Yes	2381 (13.3)	31 (1.5)	940 (19.4)	1410 (12.9)		

Lung disease	No	14,832 (82.9)	1760 (82.6)	4008 (82.9)	9064 (82.9)	0.9	0.01
	Yes	3061 (17.1)	370 (17.4)	827 (17.1)	1864 (17.1)		

Self‐rated health	Very poor	3841 (21.5)	224 (10.5)	814 (16.8)	2803 (25.6)	< 0.001	0.4
	Poor	6659 (37.2)	842 (39.5)	1445 (29.9)	4372 (40.0)		
	Average	5342 (29.9)	887 (41.6)	1583 (32.7)	2872 (26.3)		
	Good	1654 (9.2)	137 (6.4)	842 (17.4)	675 (6.2)		
	Excellent	397 (2.2)	40 (1.9)	151 (3.1)	206 (1.9)		

Depression	No	11,586 (64.8)	1088 (51.1)	3806 (78.7)	6692 (61.2)	< 0.001	0.4
	Yes	6307 (35.2)	1042 (48.9)	1029 (21.3)	4236 (38.8)		

Total recall score (median [Q1, Q3])		8.0 [5.0, 10.0]	7.0 [5.0, 9.0]	9.0 [6.0, 11.0]	8.0 [5.0, 10.0]	< 0.001	0.3

Immediate recall score (median [Q1, Q3])		5.0 [3.0, 6.0]	4.0 [3.0, 5.0]	5.0 [4.0, 6.0]	5.0 [3.0, 6.0]	< 0.001	0.3

Delayed recall score (median [Q1, Q3])		3.0 [2.0, 5.0]	3.0 [2.0, 4.0]	4.0 [3.0, 5.0]	3.0 [2.0, 5.0]	< 0.001	0.3

Orientation score (median [Q1, Q3])		4.0 [3.0, 4.0]	3.0 [2.0, 4.0]	4.0 [3.0, 4.0]	4.0 [4.0, 4.0]	< 0.001	0.5

Serial subtraction score (median [Q1, Q3])		4.0 [3.0, 5.0]	4.0 [2.0, 5.0]	4.0 [2.0, 5.0]	5.0 [3.0, 5.0]	< 0.001	0.3

Current smoker	No	15,322 (85.6)	1635 (76.8)	4231 (87.5)	9456 (86.5)	< 0.001	0.2
	Yes	2571 (14.4)	495 (23.2)	604 (12.5)	1472 (13.5)		

Alcohol consumption	No	11,139 (62.3)	1637 (76.9)	2608 (53.9)	6894 (63.1)	< 0.001	0.3
	Yes	6754 (37.7)	493 (23.1)	2227 (46.1)	4034 (36.9)		

Vigorous activity frequency (median [Q1, Q3])		3.0 [0.0, 5.0]	0.0 [0.0, 2.0]	0.0 [0.0, 1.0]	5.0 [3.0, 5.0]	< 0.001	1.5

Moderate activity frequency (median [Q1, Q3])		2.0 [2.0, 3.0]	1.0 [0.0, 7.0]	2.0 [0.0, 3.0]	2.0 [2.0, 4.0]	< 0.001	0.5

Dressing difficulty	No	14,865 (83.1)	1943 (91.2)	3856 (79.8)	9066 (83.0)	< 0.001	0.2
	Yes	3028 (16.9)	187 (8.8)	979 (20.2)	1862 (17.0)		

Bathing difficulty	No	15,199 (84.9)	1878 (88.2)	4040 (83.6)	9281 (84.9)	< 0.001	0.1
	Yes	2694 (15.1)	252 (11.8)	795 (16.4)	1647 (15.1)		

Bed difficulty	No	16,042 (89.7)	1939 (91.0)	4190 (86.7)	9913 (90.7)	< 0.001	0.1
	Yes	1851 (10.3)	191 (9.0)	645 (13.3)	1015 (9.3)		

Toilet difficulty	No	16,135 (90.2)	1718 (80.7)	4197 (86.8)	10,220 (93.5)	< 0.001	0.3
	Yes	1758 (9.8)	412 (19.3)	638 (13.2)	708 (6.5)		

Working	No	14,259 (79.7)	1207 (56.7)	3782 (78.2)	9270 (84.8)	< 0.001	0.4
	Yes	3634 (20.3)	923 (43.3)	1053 (21.8)	1658 (15.2)		

Retired	No	6243 (35.8)	1611 (75.6)	1948 (44.3)	2684 (24.6)	< 0.001	0.8
	Yes	11,214 (64.2)	519 (24.4)	2451 (55.7)	8244 (75.4)		

Social participation	No	11,148 (62.3)	1114 (52.3)	2865 (59.3)	7169 (65.6)	< 0.001	0.2
	Yes	6745 (37.7)	1016 (47.7)	1970 (40.7)	3759 (34.4)		

Hypertension medication	No	6577 (36.8)	1268 (59.5)	1320 (27.3)	3989 (36.5)	< 0.001	0.5
	Yes	11,316 (63.2)	862 (40.5)	3515 (72.7)	6939 (63.5)		

Diabetes medication	No	14,073 (78.7)	1945 (91.3)	3463 (71.6)	8665 (79.3)	< 0.001	0.3
	Yes	3820 (21.3)	185 (8.7)	1372 (28.4)	2263 (20.7)		

Survival status	No	11,863 (66.3)	1711 (80.3)	2407 (49.8)	7745 (70.9)	< 0.001	0.4
	Yes	6030 (33.7)	419 (19.7)	2428 (50.2)	3183 (29.1)		

*Note:* Continuous variables are expressed as medians with interquartile ranges [Q1, Q3], while categorical variables are reported as counts with corresponding percentages (*n* [percentage]). Statistical comparisons were performed using the Kruskal–Wallis test for continuous data and the chi‐square test for categorical variables.

Abbreviations: BMI, body mass index; CHARLS, the China Health and Retirement Longitudinal Study; HRS, the Health and Retirement Study; SHARE, the Survey of Health, Ageing and Retirement in Europe; SMD, standard mean difference.

Physical characteristics varied significantly across cohorts. Patients from CHARLS had lower BMI (median 24.5 kg/m^2^) compared to HRS (28.15 kg/m^2^) and SHARE (27.06 kg/m^2^) (*p* < 0.001). Similarly, height and weight measurements were lowest in CHARLS participants. However, grip strength was comparable across the three databases, with median values ranging from 26.0 kg (SHARE) to 28.5 kg (CHARLS) (*p* < 0.001, but with a small standardized mean difference of 0.097).

Comorbidity profiles showed notable differences. The prevalence of stroke was lowest in CHARLS (5.4%) compared to SHARE (15.6%) and HRS (18.5%) (*p* < 0.001). Similar patterns were observed for hypertension (52.3% in CHARLS, 66.6% in SHARE, and 75.6% in HRS; *p* < 0.001), diabetes (13.8% in CHARLS, 26.2% in SHARE, and 32.8% in HRS; *p* < 0.001), and cancer (1.5% in CHARLS, 12.9% in SHARE, and 19.4% in HRS; *p* < 0.001). Nevertheless, the prevalence of lung disease was consistent across all three databases (approximately 17%; *p* = 0.940).

Self‐reported health status and mental health also differed significantly. Depression was most prevalent in CHARLS (48.9%), followed by SHARE (38.8%) and HRS (21.3%) (*p* < 0.001). Cognitive function, measured by immediate recall, delayed recall, orientation, and serial subtraction scores, was generally better in HRS and SHARE participants compared to CHARLS (all *p* < 0.001).

Lifestyle behaviors showed significant variations. Current smoking was more common in CHARLS (23.2%) than in SHARE (13.5%) and HRS (12.5%) (*p* < 0.001). Conversely, alcohol consumption was more prevalent in HRS (46.1%) and SHARE (36.9%) compared to CHARLS (23.1%) (*p* < 0.001). Physical activity patterns also differed substantially, with SHARE participants reporting the highest frequency of vigorous activity (*p* < 0.001).

Functional status assessment revealed that CHARLS participants generally had fewer difficulties with activities of daily living, except for toilet difficulty, which was highest in CHARLS (19.3%) compared to HRS (13.2%) and SHARE (6.5%) (*p* < 0.001).

Socioeconomic factors varied significantly across cohorts. Working status was much higher in CHARLS (43.3%) compared to HRS (21.8%) and SHARE (15.2%) (*p* < 0.001). Conversely, retirement rates were highest in SHARE (75.4%), followed by HRS (55.7%) and CHARLS (24.4%) (*p* < 0.001). Social participation was highest in CHARLS (47.7%) and lowest in SHARE (34.4%) (*p* < 0.001).

Medication use patterns differed significantly. Hypertension medication use was lowest in CHARLS (40.5%) compared to SHARE (63.5%) and HRS (72.7%) (*p* < 0.001). Similarly, diabetes medication use was lowest in CHARLS (8.7%) compared to SHARE (20.7%) and HRS (28.4%) (*p* < 0.001). Further detailed comparisons of all variables between the three databases are provided in Supporting Information 4: Table S1.

### 3.2. Feature Selection and Model Development

Feature selection using the Boruta algorithm identified varying numbers of important predictors across the three databases (Figure 2). In the SHARE cohort, 34 features were confirmed as important, with one feature (ever smoked) classified as tentatively important. The most significant features based on mean importance scores were age (63.03), moderate activity (30.45), bathing difficulty (24.11), total recall (23.50), and self‐rated health (22.08) (Figure 2a). In the HRS cohort, 34 features were confirmed as important, with three features (ever smoked, arthritis, and currently smoking) classified as tentatively important. The most significant features were age (59.22), grip strength (31.97), working (24.49), moderate activity (22.68), and total recall (21.90) (Figure 2b). The CHARLS cohort had 25 confirmed important features and four tentatively important features (toilet difficulty, drinking, currently smoking, and self‐rated memory). The most significant features were age (34.51), vigorous activity (22.10), moderate activity (13.54), bathing difficulty (12.40), and working (12.28) (Figure 2c).

Figure 2Feature importance distribution based on Boruta analysis. This figure displays the distribution of feature importance *Z*‐scores from Boruta analysis for heart disease mortality prediction. (a) SHARE database, (b) HRS database, and (c) CHARLS database. Features are ordered by mean importance, with color coding for confirmed important (blue), tentatively important (light blue), and rejected (gray) features. The vertical axis represents features, and the horizontal axis represents the feature importance *Z*‐score distribution. Abbreviations: SHARE, the Survey of Health, Ageing and Retirement in Europe; HRS, the Health and Retirement Study; CHARLS, the China Health and Retirement Longitudinal Study; BMI, body mass index.

**Figure:**
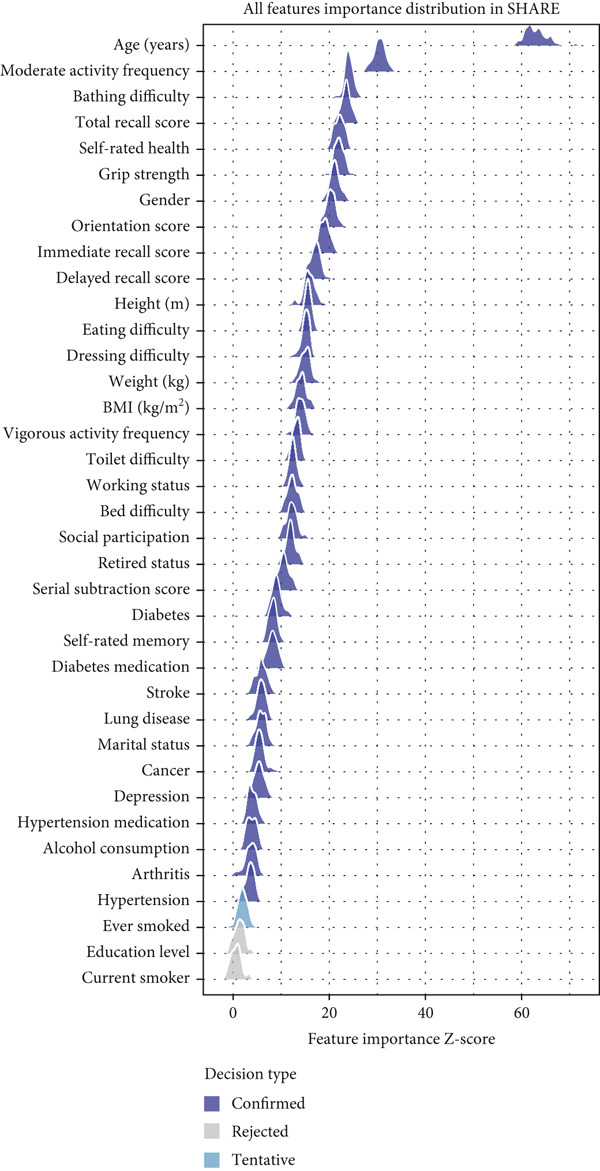
(a)

**Figure:**
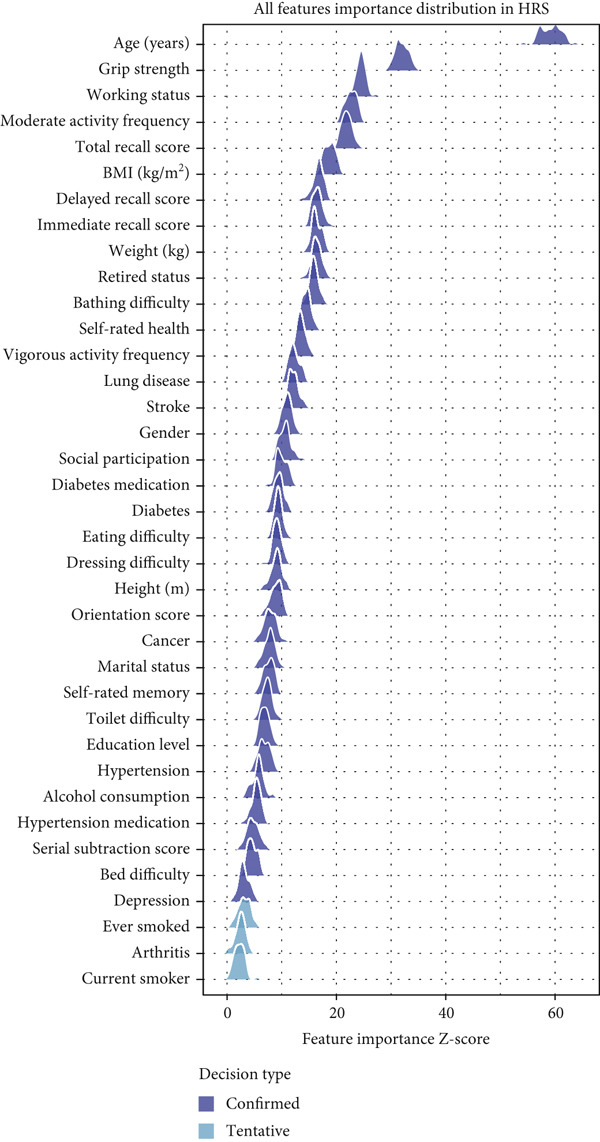
(b)

**Figure:**
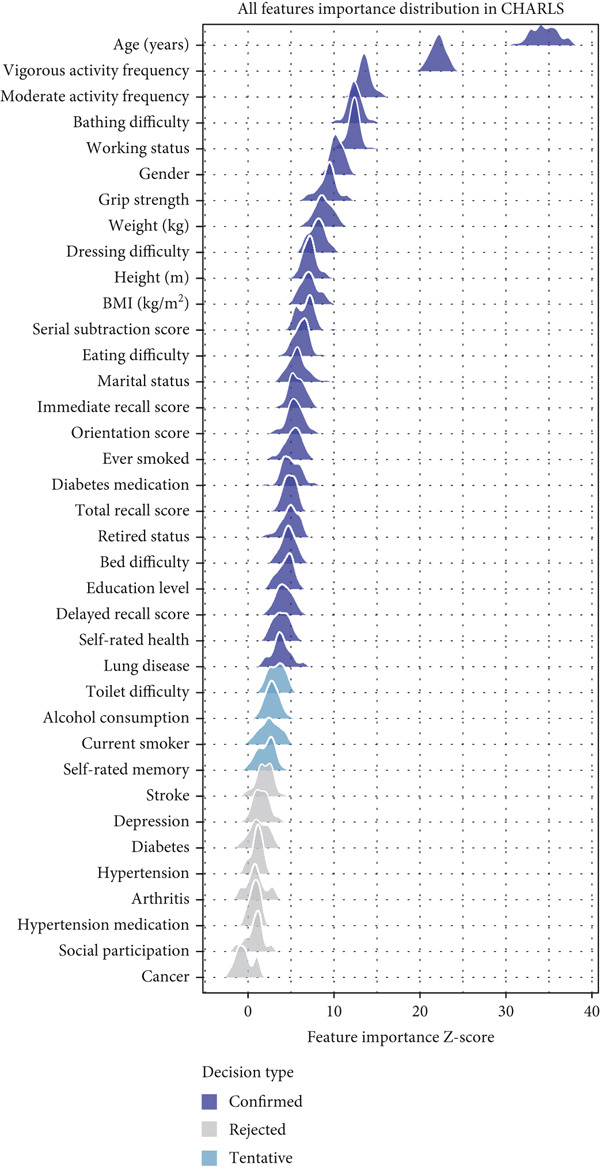
(c)

We used fivefold cross‐validation with three repetitions (a total of 15 validations) to ensure the stability of parameter selection. XGBoost′s performance remained stable across all datasets (AUC range: 0.770–0.821 as shown in Table 2), indicating successful hyperparameter tuning. We adopted stratified *k*‐fold cross‐validation to ensure consistent event rates across each fold and reduced the impact of randomness by performing repeated cross‐validation (three repetitions). To develop a robust cross‐cohort prediction model, we identified the common set of features that were important across all three databases. This resulted in 27 common predictors: age, gender, marital status, diabetes med, lung disease, self‐rated health, self‐rated memory, dressing difficulty, bathing difficulty, eating difficulty, bed difficulty, toilet difficulty, immediate recall, delayed recall, total recall, serial subtraction, orientation, ever smoked, drinking, vigorous activity, moderate activity, height, weight, BMI, grip strength, retired, and working. These common predictors encompassed demographic characteristics, physical measurements, chronic conditions, functional status, cognitive function, lifestyle behaviors, and socioeconomic factors, representing a comprehensive set of risk markers for heart disease mortality.

The consistency of these 27 predictors across three distinct aging cohorts from different regions (Europe, United States, and China) suggests their robust association with heart disease mortality regardless of geographic, ethnic, or healthcare system differences. These common features were used as inputs for subsequent ML model development to ensure model generalizability across diverse populations.

### 3.3. ML Model Performance

Using the 27 common features identified across all three cohorts, we developed and evaluated 11 different ML models for predicting heart disease mortality. Figure 3 presents the ROC curves for all models across different datasets, with performance metrics summarized in Table 4 and visualized as a heat map in Figure 3e.

Figure 3ROC curves and AUC heatmap for heart disease mortality prediction models. This figure presents ROC curves for heart disease mortality prediction models across different datasets: (a) SHARE training set, (b) SHARE internal validation set, (c) HRS external validation set, and (d) CHARLS external validation set. Each curve represents a different machine learning model, with the area under the curve (AUC) value included in the legend. (e) Heatmap visualization of AUC values for each model across datasets, with color intensity indicating AUC magnitude. Abbreviations: SHARE, the Survey of Health, Ageing and Retirement in Europe; HRS, the Health and Retirement Study; CHARLS, the China Health and Retirement Longitudinal Study; ROC, receiver operating characteristic; XGBoost, the extreme gradient boosting; AdaBoost, the adaptive boosting; GBM, gradient boosting machine; KNNs, *k*‐nearest neighbors; SVM, support vector machine; CARTs, classification and regression trees.

**Figure:**
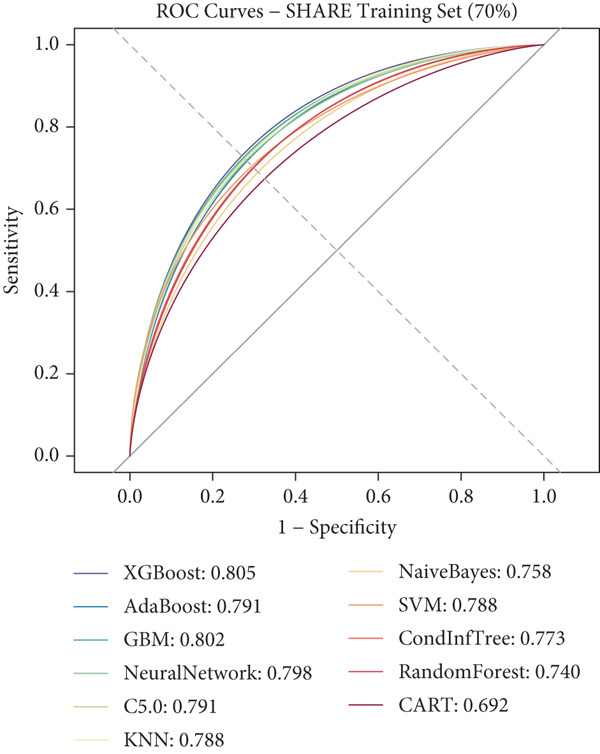
(a)

**Figure:**
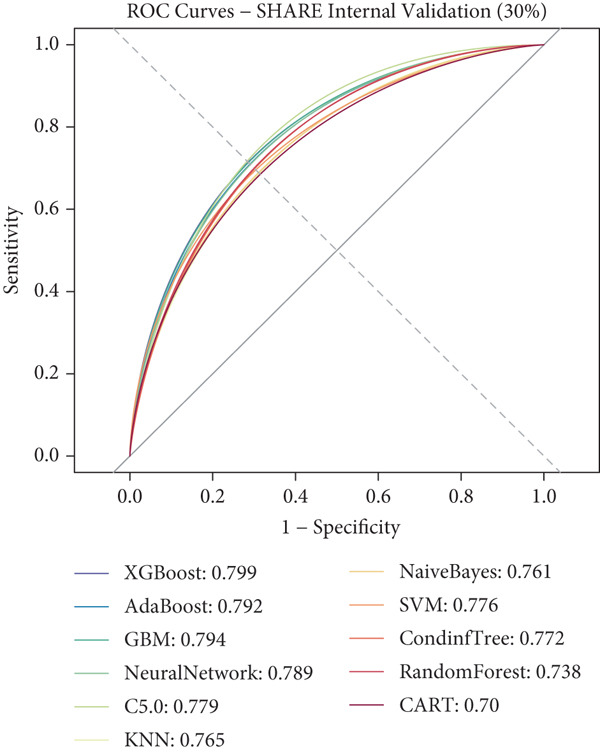
(b)

**Figure:**
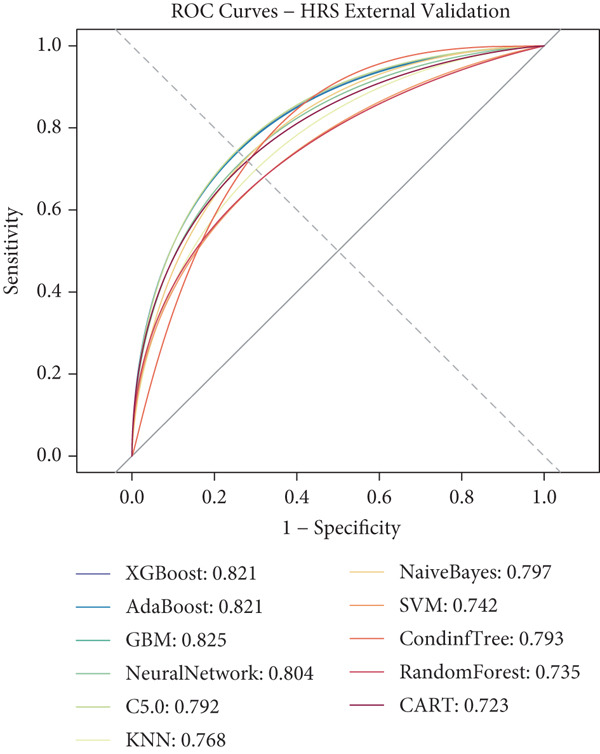
(c)

**Figure:**
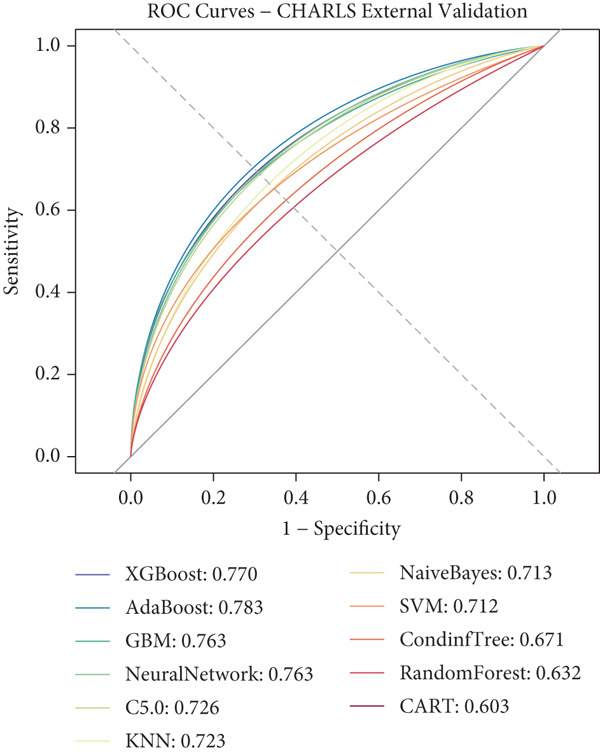
(d)

**Figure:**
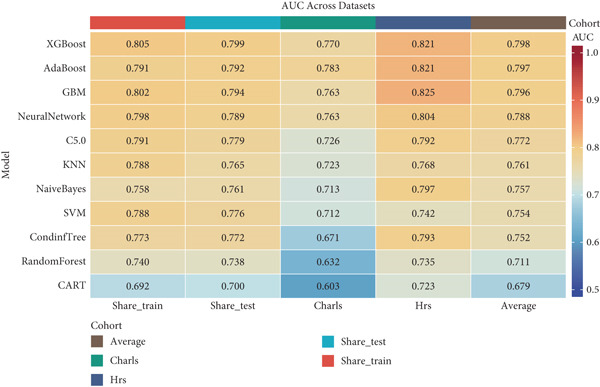
(e)

**Table 4:** Performance summary of machine learning models for heart disease mortality prediction.

**Model**	**Dataset**	**AUC**	**CI_Lower**	**CI_Upper**	**Optimal_Threshold**	**Sensitivity**	**Specificity**	**Youden_Index**
RandomForest	SHARE_Train	0.74	0.73	0.75	0.05	0.64	0.73	0.38
RandomForest	SHARE_Test	0.74	0.72	0.76	0.05	0.65	0.73	0.38
RandomForest	CHARLS	0.63	0.60	0.66	0.09	0.45	0.79	0.23
RandomForest	HRS	0.74	0.72	0.75	0.01	0.70	0.70	0.40
XGBoost	SHARE_Train	0.81	0.79	0.82	0.27	0.76	0.70	0.46
XGBoost	SHARE_Test	0.80	0.78	0.82	0.28	0.74	0.72	0.46
XGBoost	CHARLS	0.77	0.74	0.80	0.14	0.72	0.69	0.41
XGBoost	HRS	0.82	0.81	0.83	0.22	0.68	0.82	0.49
SVM	SHARE_Train	0.79	0.78	0.80	0.23	0.73	0.73	0.46
SVM	SHARE_Test	0.78	0.76	0.79	0.23	0.70	0.74	0.45
SVM	CHARLS	0.71	0.68	0.74	0.22	0.63	0.69	0.33
SVM	HRS	0.74	0.73	0.76	0.22	0.62	0.73	0.36
NeuralNetwork	SHARE_Train	0.80	0.79	0.81	0.26	0.76	0.69	0.45
NeuralNetwork	SHARE_Test	0.79	0.77	0.81	0.28	0.73	0.72	0.45
NeuralNetwork	CHARLS	0.76	0.74	0.79	0.14	0.67	0.72	0.39
NeuralNetwork	HRS	0.80	0.79	0.82	0.19	0.67	0.80	0.48
GBM	SHARE_Train	0.80	0.79	0.81	0.29	0.72	0.74	0.46
GBM	SHARE_Test	0.79	0.78	0.81	0.31	0.68	0.76	0.44
GBM	CHARLS	0.76	0.74	0.79	0.15	0.71	0.69	0.41
GBM	HRS	0.83	0.81	0.84	0.19	0.73	0.78	0.50
NaiveBayes	SHARE_Train	0.76	0.75	0.77	0.00	0.64	0.74	0.38
NaiveBayes	SHARE_Test	0.76	0.74	0.78	0.00	0.72	0.68	0.40
NaiveBayes	CHARLS	0.71	0.69	0.74	0.00	0.72	0.61	0.33
NaiveBayes	HRS	0.80	0.78	0.81	0.00	0.78	0.66	0.44
KNN	SHARE_Train	0.79	0.78	0.80	0.24	0.77	0.65	0.42
KNN	SHARE_Test	0.77	0.75	0.78	0.31	0.68	0.73	0.41
KNN	CHARLS	0.72	0.70	0.75	0.23	0.63	0.73	0.36
KNN	HRS	0.77	0.76	0.78	0.15	0.71	0.69	0.40
CART	SHARE_Train	0.69	0.68	0.70	0.26	0.61	0.73	0.34
CART	SHARE_Test	0.70	0.68	0.72	0.26	0.61	0.74	0.36
CART	CHARLS	0.60	0.58	0.62	0.26	0.25	0.96	0.21
CART	HRS	0.72	0.71	0.74	0.26	0.57	0.85	0.42
AdaBoost	SHARE_Train	0.79	0.78	0.80	0.36	0.71	0.74	0.45
AdaBoost	SHARE_Test	0.79	0.78	0.81	0.38	0.66	0.78	0.44
AdaBoost	CHARLS	0.78	0.76	0.81	0.21	0.78	0.67	0.45
AdaBoost	HRS	0.82	0.81	0.83	0.30	0.66	0.83	0.49
C5.0	SHARE_Train	0.79	0.78	0.80	0.21	0.69	0.76	0.45
C5.0	SHARE_Test	0.78	0.76	0.80	0.18	0.73	0.73	0.45
C5.0	CHARLS	0.73	0.70	0.75	0.07	0.69	0.69	0.38
C5.0	HRS	0.79	0.78	0.81	0.08	0.71	0.78	0.48
CondInfTree	SHARE_Train	0.77	0.76	0.79	0.32	0.64	0.78	0.42
CondInfTree	SHARE_Test	0.77	0.76	0.79	0.26	0.73	0.70	0.42
CondInfTree	CHARLS	0.67	0.64	0.70	0.21	0.54	0.81	0.35
CondInfTree	HRS	0.79	0.78	0.81	0.23	0.65	0.81	0.46

*Note:* This table provides a comparative summary of the performance metrics for 11 machine learning algorithms evaluated across three datasets: training, internal validation, and external validation. Reported metrics encompass the area under the receiver operating characteristic curve (AUC) with corresponding 95% confidence intervals, as well as sensitivity, specificity, and overall accuracy. Models are ordered by their average AUC across all datasets.

Abbreviations: AdaBoost, the adaptive boosting; CARTs, classification and regression trees; CHARLS, the China Health and Retirement Longitudinal Study; GBM, gradient boosting machine; HRS, the Health and Retirement Study; KNNs, *k*‐nearest neighbors; SHARE, the Survey of Health, Ageing and Retirement in Europe; SVM, support vector machine; XGBoost, the extreme gradient boosting.

XGBoost demonstrated the best overall performance with the highest average AUC of 0.798 across all datasets. In the SHARE training set, XGBoost achieved an AUC of 0.805 (95% CI: 0.794–0.815) with a sensitivity of 0.755 and specificity of 0.702 at the optimal threshold of 0.271. In the SHARE internal validation set, the model preserved robust performance with an AUC of 0.799 (95% CI: 0.782–0.815), showing a difference of only 0.006 (0.745%) between training and validation sets, well below the 5% threshold that would indicate overfitting. This confirms the model′s excellent internal validity and stability.

More impressively, XGBoost maintained strong performance in external validation, achieving a high AUC of 0.821 (95% CI: 0.809–0.833) in the HRS dataset and 0.770 (95% CI: 0.744–0.795) in the CHARLS dataset. The model′s ability to maintain and even slightly improve performance in the HRS dataset compared to the training dataset demonstrates exceptional generalizability across different populations. The optimal prediction threshold varied across datasets (from 0.143 in CHARLS to 0.284 in the SHARE testing set), reflecting the differing baseline mortality rates, yet the model maintained balanced sensitivity and specificity across diverse populations.

While other ensemble methods like AdaBoost (average AUC: 0.797) and GBM (average AUC: 0.796) also performed well, and traditional algorithms showed relatively lower performance, XGBoost consistently demonstrated superior predictive capability and generalizability. This robust performance across datasets with different ethnic, cultural, and healthcare backgrounds suggests XGBoost′s potential as a reliable risk stratification tool for heart disease mortality prediction in elderly populations worldwide.

The performance differences across datasets are mainly due to variations in population characteristics and event rates (Table 2): HRS performed best (AUC = 0.8211): The event rate of 50.2% is close to perfect balance, and the US population may have more complete medical records. SHARE internal validation (AUC = 0.7988): Consistent with the training set, the performance is stable. CHARLS relatively lower (AUC = 0.7695): The event rate is only 19.7%, resulting in class imbalance; disease patterns in the Asian population may differ from those in Europe, and the US calibration analysis supports this explanation: CHARLS′s E/O ratio is 0.7829, indicating that the model tends to overestimate the risk, which is a common phenomenon in low event rate populations.

The GRACE score requires specific clinical variables (such as troponin, creatinine levels, Killip classification, and other acute coronary syndrome–related indicators), which are not available in our population health survey data. Therefore, we chose to compare with the Charlson Index and the Framingham score, which are more suitable for general populations. These two scoring systems have been widely validated in community populations and are more aligned with our research context. The comparison of our model with the two scoring systems is detailed in Table 2. When compared with the Charlson Comorbidity Index, XGBoost outperformed the Charlson Index in all datasets (SHARE: 0.799 vs. 0.728, *p* < 0.001) and indicated significant improvement in risk reclassification (NRI = 0.190 and IDI = 0.209). When compared with the Framingham Risk Score, XGBoost performed significantly better (SHARE: 0.799 vs. 0.663, *p* < 0.001), and the advantage remained consistent across all external validation datasets.

### 3.4. Model Calibration Evaluation and Decision Curve Analysis (DCA)

Calibration metrics have been fully reported: Brier score: SHARE training set: 0.1555 (excellent), SHARE test set: 0.1588 (excellent), CHARLS: 0.1351 (excellent), and HRS: 0.2585 (acceptable). Hosmer–Lemeshow test: SHARE test set: *χ* ^2^ = 22.53, *p* = 0.004; CHARLS: *χ* ^2^ = 51.62, *p* < 0.001; and HRS: *χ* ^2^ = 2229.92, *p* < 0.001. Calibration slope and intercept: SHARE test set: slope = 1.149, intercept = 0.117 (close to ideal values of 1 and 0); CHARLS: slope = 1.279, intercept = 0.768 (requires recalibration), and HRS: slope = 1.518, intercept = 2.160 (significantly deviates, reflecting population differences). The E/O ratio provide the expected/observed mortality ratio. SHARE is close to 1.0 (0.996), indicating good calibration. We evaluated clinical net benefit through NRI and IDI analysis: with −0.154 of the NRI and 0.009 of the IDI for SHARE validation set, indicating slight improvement in probability prediction. The comparison with traditional scores provided clinical value evidence (Table 2): NRI > 0 in all datasets compared to the Charlson Index (SHARE: 0.190, CHARLS: 0.335, and HRS: 0.329) and AUC improvement is significant compared to the Framingham score (SHARE: 0.799 vs. 0.663).

### 3.5. Model Interpretability via SHAP Analysis

To facilitate the transparency and interpretability of the XGBoost model, we employed SHAP analysis to identify key features driving mortality predictions across all cohorts. Figure 4 presents the Top 10 predictors ranked by their mean absolute SHAP values across different datasets. Age consistently emerged as the most influential predictor across all cohorts, with mean SHAP values of 0.101 in SHARE training (Figure 4a), 0.099 in SHARE testing (Figure 4b), 0.102 in HRS (Figure 4c), and 0.056 in CHARLS (Figure 4d).

Figure 4Top 10 features by importance based on SHAP analysis. This figure shows the Top 10 features by importance based on SHAP analysis for heart disease mortality prediction: (a) SHARE training set, (b) SHARE testing set, (c) HRS dataset, and (d) CHARLS dataset. Features are ranked by their mean absolute SHAP values, which quantify their contribution to the model predictions. The color gradient represents the feature importance magnitude. Abbreviations: SHARE, the Survey of Health, Ageing and Retirement in Europe; HRS, the Health and Retirement Study; CHARLS, the China Health and Retirement Longitudinal Study; SHAP, SHapley Additive exPlanations; XGBoost, the extreme gradient boosting.

**Figure:**
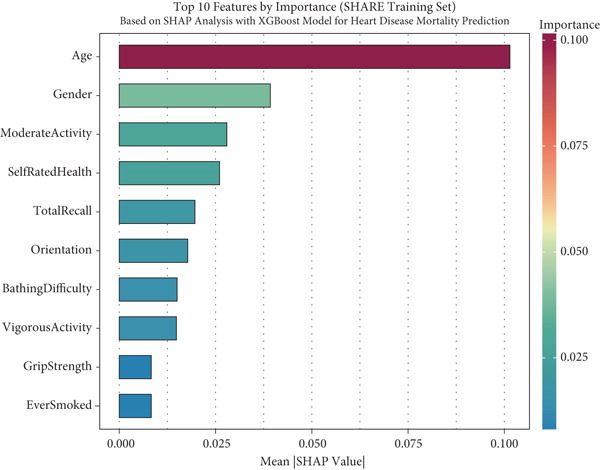
(a)

**Figure:**
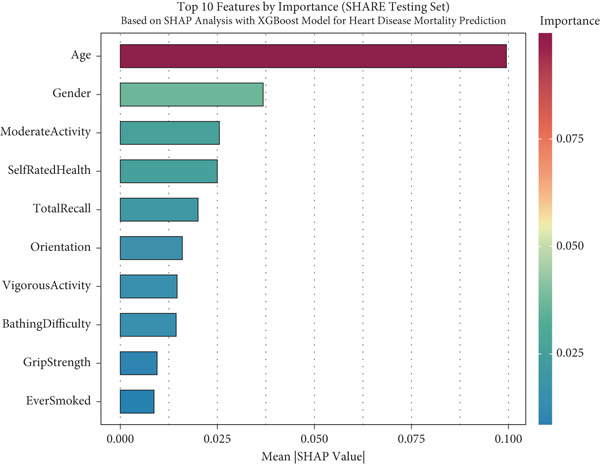
(b)

**Figure:**
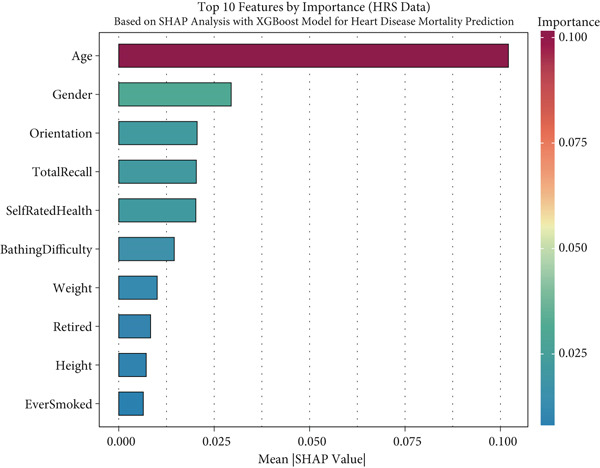
(c)

**Figure:**
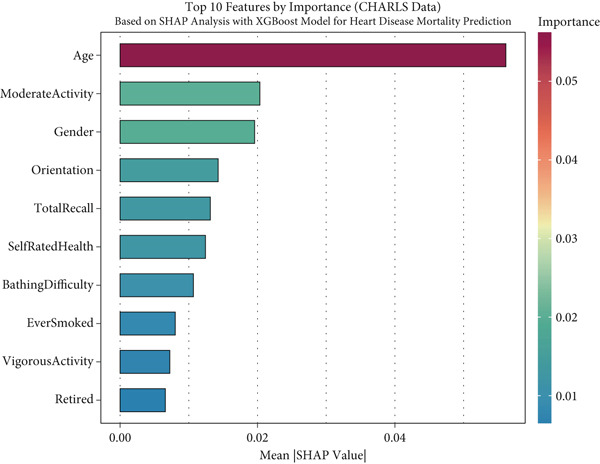
(d)

Interestingly, the ranking of other predictors varied across cohorts, reflecting population‐specific risk factors. In the SHARE training and testing datasets, the Top 5 predictors maintained the same order: age, gender (0.039 and 0.037), moderate activity (0.028 and 0.026), self‐rated health (0.026 and 0.025), and total recall (0.020 and 0.020). However, the pattern differed in the CHARLS dataset, where moderate activity (0.020) ranked second, followed by gender (0.020), orientation (0.014), and total recall (0.013), with self‐rated health (0.012) ranking sixth. In the HRS dataset, gender (0.029) ranked second but was followed by orientation (0.021), total recall (0.020), and self‐rated health (0.020). These variations likely reflect differences in sociodemographic characteristics, healthcare systems, and cultural contexts across the three regions, influencing how specific factors contribute to mortality risk. Complete SHAP summary plots for all features are provided in Supporting Information 1: Figures S1–S4, showing the pattern of SHAP values for all features in the model across the SHARE training set (Supporting Information 1: Figure S1), SHARE testing set (Supporting Information 1: Figure S2), HRS dataset (Supporting Information 1: Figure S3), and CHARLS dataset (Supporting Information 1: Figure S4). These comprehensive visualizations further support the consistent patterns of feature importance across datasets while highlighting the cohort‐specific variations in magnitude.

**Figure 5:**
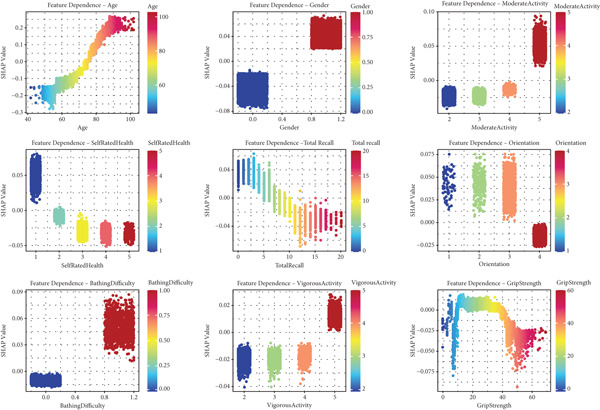
SHAP feature dependence plots for SHARE training set. This figure displays the relationship between feature values and their SHAP values for the Top 9 important features in the SHARE training set. Each point represents an individual instance, with color indicating the feature value. The vertical axis shows the SHAP value (impact on model output), and the horizontal axis shows the feature value. These plots reveal how the model predictions change as a function of the input features. Abbreviation: SHAP, SHapley Additive exPlanations.

The feature dependence plots revealed important insights into how each predictor influenced mortality risk (Figure 5). Age demonstrated a strong positive relationship with mortality risk, with SHAP values increasing almost linearly from age 40 to 100, suggesting that mortality risk progressively increases with age. Gender showed a clear dichotomous effect, with male gender (coded as 1) associated with higher mortality risk (positive SHAP values) and female gender (coded as 0) with lower risk (negative SHAP values). Self‐rated health exhibited an inverse relationship with mortality, where excellent health (Rating 1) was associated with increased survival (negative SHAP values), while poor health (Ratings 4–5) predicted higher mortality risk (positive SHAP values).

Moderate activity, total recall, and orientation also showed important relationships with mortality risk as seen in Figure 5. Functional and physical performance measures revealed nonlinear relationships with mortality. Bathing difficulty (coded as 1) was strongly associated with increased mortality risk, while vigorous physical activity frequency showed a dose–response relationship, with higher frequency (Level 5) associated with decreased mortality risk. Grip strength demonstrated a complex U‐shaped relationship, with both insufficient and excessive levels associated with elevated mortality risk, although the relationship was stronger at lower values.

Additionally, feature dependence plots for the external validation datasets are presented in Supporting Information 2: Figures S5–S7, displaying how SHAP values change with feature values in the SHARE testing set (Supporting Information 2: Figure S5), HRS dataset (Supporting Information 2: Figure S6), and CHARLS dataset (Supporting Information 2: Figure S7). These plots demonstrate remarkable consistency in the directional relationships between features and mortality risk across different populations, despite variations in the magnitude of effects.

Despite differences in the ranking and magnitude of SHAP values across datasets, the directional relationships between features and mortality risk remained remarkably consistent. This cross‐cohort consistency validates the biological plausibility of these predictors while acknowledging the cultural and healthcare system differences that may influence the relative importance of specific risk factors in different populations. Such insights could inform the development of culturally tailored interventions and facilitate the global scalability of personalized CVD risk prediction tools.

### 3.6. Individual‐Level Risk Explanation and Clinical Interpretation

To provide clinical context to our predictive model, we examined how features collectively influence predictions for individual patients through SHAP force plots and waterfall comparisons. Figures 6a, 6b, 6c, and 6d present force plots across all four datasets, and these visualizations illustrate the heterogeneity in risk profiles across patients, with age consistently exerting the strongest influence across most individuals.

Figure 6SHAP force plots and waterfall comparison for heart disease mortality prediction. This figure depicts SHAP force plots showing feature contributions to mortality predictions: (a) SHARE training set, (b) SHARE testing set, (c) HRS dataset, and (d) CHARLS dataset. (e) Comparison of SHAP waterfall plots for representative high‐risk (left) and low‐risk (right) heart disease patients from the SHARE training set showing how individual features contribute to the prediction. Abbreviations: SHARE, the Survey of Health, Ageing and Retirement in Europe; HRS, the Health and Retirement Study; CHARLS, the China Health and Retirement Longitudinal Study; XGBoost, the extreme gradient boosting; SHAP, SHapley Additive exPlanations.

**Figure:**
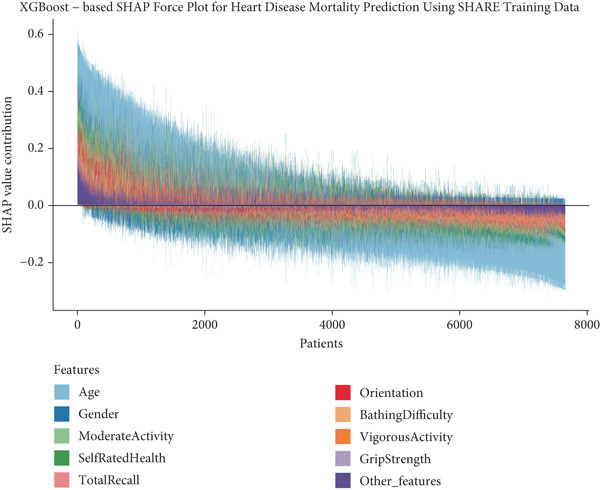
(a)

**Figure:**
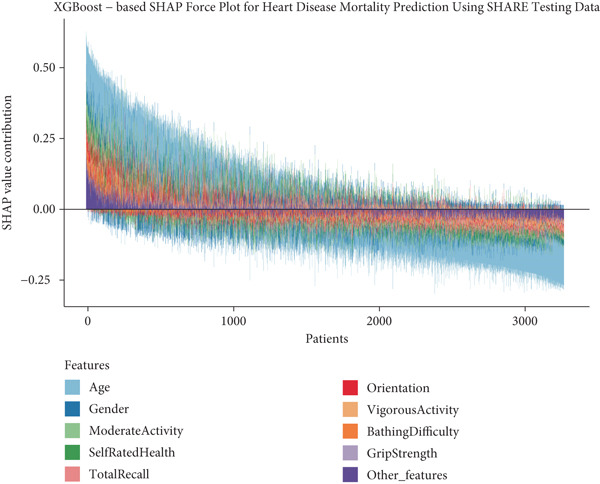
(b)

**Figure:**
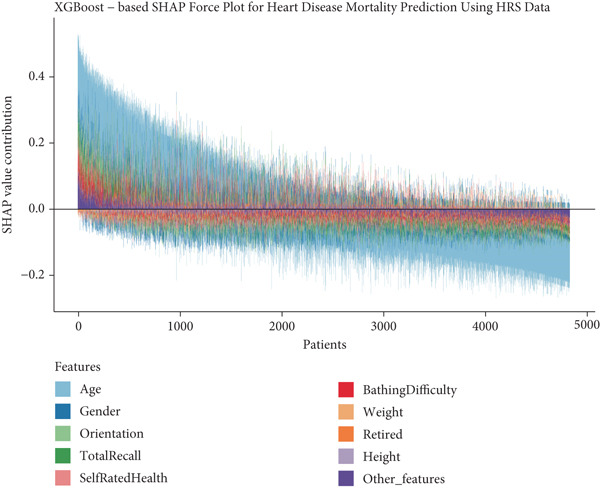
(c)

**Figure:**
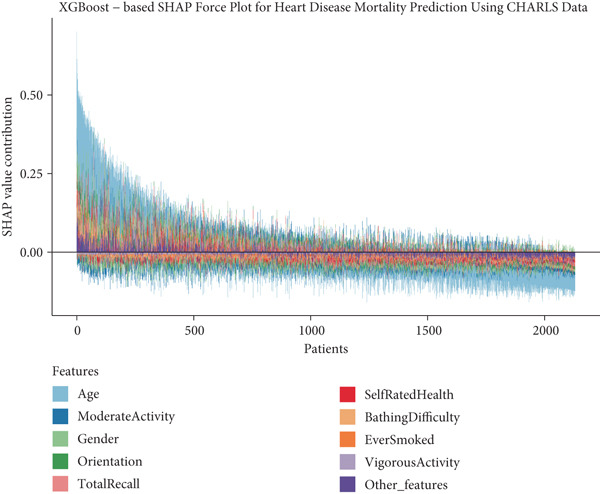
(d)

**Figure:**
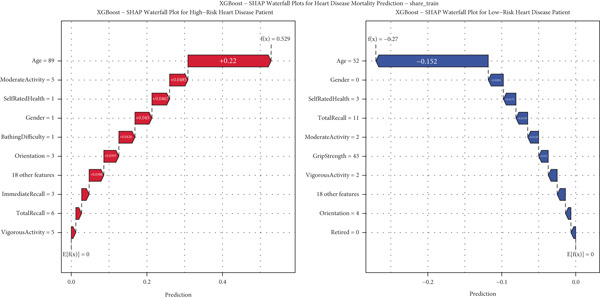
(e)

For detailed clinical interpretation, we compared representative high‐risk and low‐risk patients through waterfall plots (Figure 6e). In the high‐risk patient from the SHARE training dataset, age (89 years) contributed most significantly to elevated risk (SHAP value: 0.2200), followed by moderate activity level (Level 5, SHAP: 0.0485), poor self‐rated health (Level 1, SHAP: 0.0467), male gender (SHAP: 0.0450), and bathing difficulty (SHAP: 0.0426). Conversely, in the low‐risk patient, younger age (52 years) was the dominant protective factor (SHAP: −0.1521), followed by better cognitive function (total recall score: 11, SHAP: −0.0159), higher grip strength (43 kg, SHAP: −0.0130), and frequent vigorous activity (SHAP: −0.0121). Interestingly, while most features aligned with expected clinical relationships, some exhibited counterintuitive effects; for instance, the high‐risk patient′s high moderate activity level (5) paradoxically increased risk, potentially reflecting compensatory behaviors in response to declining health.

Similar patterns emerged in the external validation datasets, with some cohort‐specific variations (Supporting Information 3: Figures S8–S10). Age remained the dominant factor across all cohorts, but the contribution of physical function measures (bathing difficulty and grip strength) varied more substantially between high and low‐risk patients in the HRS dataset (Supporting Information 3: Figure S9) compared to the CHARLS dataset (Supporting Information 3: Figure S10), potentially reflecting differences in functional assessment or cultural interpretations of disability.

These individual‐level explanations demonstrate how the model integrates multiple risk factors to generate personalized risk assessments, potentially enabling more targeted interventions. The consistency of risk factor patterns across diverse populations affirms the model′s capacity to characterize fundamental biological mechanisms of mortality risk while accommodating population‐specific variations in risk factor distribution and impact.

The calibration metric report is calculated. The data of the SHARE test set are as follows: slope = 1.149, intercept = 0.117, Brier = 0.159, and H‐L *p* = 0.004. The CHARLS set: slope = 1.279, intercept = 0.768, Brier = 0.135, and H‐L *p* < 0.001. The HRS et: slope = 1.518, intercept = 2.160, Brier = 0.259, and H‐L *p* < 0.001. And the E/O ratio is 0.996 (excellent), 0.783 (needs adjustment upward), and 0.474 (needs adjustment upward).

### 3.7. Subgroup Analysis

Age‐stratified analysis hint model performance declines with increasing age, reflecting the increased complexity of predictions for the elderly group. The model maintained good performance in all age groups (AUC > 0.62), demonstrating that it captured important information beyond age. Gender and comorbidity stratification is detailed in Table 1. The mortality rate of men was higher in SHARE (56.7% vs. 43.3%) and CHARLS (51.6% vs. 48.4%). In the SHARE dataset, the mortality rates of patients with diabetes and hypertension were 31.8% and 70.8%. In addition, multiple ADL difficulties significantly increase the risk of death (e.g., the mortality rate for those with difficulty in dressing is 28.4% vs. 71.6% for those without difficulty).

The model performance after removing age is calculated. The AUC values decreased by 9.5%, 16.9%, and 11.5%, respectively, for the SHARE validation set, CHARLS set, and HRS set.

## 4. Discussion

In this large‐scale, multinational cohort study, we developed and externally validated an interpretable ML model to predict all‐cause mortality in elderly patients with heart disease using harmonized data from three major longitudinal aging studies: CHARLS (China), SHARE (Europe), and HRS (United States). Our XGBoost‐based model demonstrated excellent internal and external performance (average AUC > 0.80), with strong generalizability across continents and diverse healthcare systems.Our findings revealed that age, gender, moderate physical activity, and self‐rated health were the most consistent and influential predictors of mortality. Notably, grip strength and physical activity also contributed meaningfully to risk prediction, with nonlinear relationships observed through SHAP analysis. These results underscore the need to consider geriatric‐specific domains when evaluating cardiovascular risk in older adults.

Based on the importance of variables and clinical data, we identified several obvious high‐risk markers and proposed actionable suggestions (Table 1). For patients with deteriorating functional status, especially those with difficulty bathing, the risk of death increases (SHARE: 29.3% vs. 70.7%). We recommend early functional assessment and rehabilitation intervention for them. For patients with cognitive decline, the median of immediate recall is 4 in the death group versus 5 in the survival group (SHARE), and the median of delayed recall is 2 in the death group versus 4 in the survival group (SHARE). We recommend regular cognitive screening and early intervention. At the same time, we suggest incorporating grip strength measurement into routine physical examinations. Those who have both diabetes and hypertension are at the highest risk. It is recommended to develop a comprehensive chronic disease management strategy as early as possible.

Previous cardiovascular risk tools such as the Framingham Risk Score and GRACE score have been widely used but are limited by their reliance on middle‐aged cohorts and focus on traditional biomedical markers [6, 21]. However, when applied to elderly patients with heart disease, these models have some well‐known flaws. These models are mainly developed based on the data of middle‐aged people, which limits their applicability to the elderly, as the risk status of the elderly is significantly different from that of the young [22]. Additionally, these models mainly focus on traditional clinical and biochemical indicators, such as blood pressure, cholesterol, and diabetes, but rarely incorporate indicators such as fattening, cognitive decline, or dysfunction. As the aging process is associated with various physiological, cognitive, and behavioral changes, relying solely on traditional biomarkers may lead to misclassification of risks and underestimation of the vulnerability of elderly patients [23, 24]. Our study diverges from prior work in several critical ways. These limitations highlight the necessity of a paradigm shift in cardiovascular risk prediction, which should take into account the complexity and multidimensionality of aging. First, one of the core innovations in our research is the adoption of a multicohort design and cross‐cultural validation, integrating data from three representative aging cohorts in Asia, Europe, and North America. The multicohort design can reduce overfitting and alleviate the inherent bias of the unit point model, thereby improving the external validity and ensuring that the obtained model is applicable to different racial, geographical, and healthcare contexts [25, 26]. For instance, the integrated ML model developed by Cheng et al. demonstrated robust performance in both internal and external validation datasets, highlighting the effectiveness of the multicohort approach in capturing the heterogeneity of elderly patients with heart disease [25]. Similarly, the comprehensive review recommendations for cardiovascular risk in the elderly should be validated across cultures to overcome the obvious limitations of traditional scores, which often have calibration errors when applied to non‐Western or highly diverse populations [22]. Our work builds on these insights and is specifically targeted at the international elderly population, ensuring that the predictive model maintains accuracy, reliability, and fairness in various clinical settings. Another key distinction of our study is the development of a coordinated feature set that incorporates a broad spectrum of predictive factors. Traditional models have primarily focused on standard biomedical markers; however, recent evidence suggests that comprehensive geriatric assessment—including evaluations of frailty, cognitive decline, functional impairment, and behavioral changes—can more accurately capture the overall vulnerability of older adults [23, 24]. Frailty, for example, is a multidimensional syndrome defined by reduced physiological reserves, and its assessment has been shown to independently estimate negative outcomes such as mortality and hospital readmission in elderly patients with CVD [27]. Our approach builds upon these findings by integrating diverse data types into a unified predictive framework, thereby addressing previously unmet clinical needs in geriatric cardiology. Third, our study employed interpretable artificial intelligence techniques, particularly the SHAP method, to elucidate the contribution of each variable to individual patient predictions through detailed visualizations. This enables clinicians to gain insights into how both global and local features influence mortality risk, thereby enhancing personalized risk communication. Wang et al. demonstrated that the use of SHAP not only improved the interpretability of their XGBoost model in estimating 3‐year all‐cause mortality in the heart failure population but also helped identify key predictors such as age, NT‐proBNP levels, and NYHA classification [28].

Age, as a nonmodifiable variable, consistently plays a central role in cardiovascular risk models, with its predictive power validated across various cohorts [29]. However, as a fundamental predictor, age alone is insufficient to comprehensively capture individual risk. Accurate risk assessment requires its integration with additional functional, behavioral, and psychological variables to achieve a more precise and personalized evaluation [30]. Consistent with the findings of most epidemiological studies, male patients with heart disease generally exhibit a higher risk of mortality compared to females [31, 32]. This disparity is largely attributed to differences in absolute risk factors, behavioral patterns, and underlying physiological mechanisms. Moderate activity is widely regarded as a key intervention for improving prognosis and is commonly recommended in the primary prevention of CVD [33–35]. However, in certain high‐risk individuals, it has been paradoxically linked to a higher likelihood of death. This may reflect the so‐called “physical activity paradox,” which can arise from multiple factors. First, in individuals with marginal functional status, physical activity may predominantly consist of involuntary or necessary labor—for example, older adults in rural areas may be compelled to engage in physically demanding tasks despite poor baseline health. Second, physical activity data are often self‐reported, introducing subjectivity and potential overestimation. Third, some patients may have underlying comorbidities or insufficient physiological reserve, limiting the protective effects of physical activity even at higher levels [35]. Hence, in clinical practice, it is crucial to avoid the oversimplified assumption that “more activity is always better” when designing interventions. An especially noteworthy finding was the U‐shaped association between grip strength and mortality, indicating that both abnormally low and excessively high values were linked to increased risk. While reduced grip strength is consistently recognized as a reliable measure of sarcopenia and frailty, unusually high values may reflect underlying pathophysiological conditions—such as compensatory overactivation—or potential measurement artifacts. While most previous studies—primarily conducted in non‐Asian populations—have suggested that higher grip strength serves as a protective factor against CVD, our findings reveal a U‐shaped relationship between grip strength and cardiovascular mortality [36–39]. This indicates that both abnormally low and excessively high grip strength values are associated with increased risk. Although reduced grip strength is widely recognized as a reliable indicator of sarcopenia and frailty, unusually high values may reflect underlying pathophysiological conditions, such as compensatory overactivation, or may represent potential measurement artifacts. Cognitive impairment predicts poor medication adherence, diminished ability to manage chronic disease, and greater risk of adverse outcomes. Self‐rated health, though subjective, integrates an individual′s perception of their overall physiological state.

Our model demonstrates strong potential for clinical integration, as all 27 input variables are routinely available in outpatient or primary care settings. The use of SHAP enhances interpretability, enabling clinicians to identify individual risk factors—such as reduced grip strength, cognitive decline, or poor self‐rated health—that are often overlooked in traditional cardiology tools. This supports personalized interventions, including early rehabilitation or advanced care planning. The model′s strong external validation suggests global applicability: integration into electronic health records in high‐income countries and simplified tools for community screening in low‐resource settings. Methodologically, our study benefits from three nationally representative aging cohorts, rigorous data preprocessing, Boruta‐based feature selection, and SHAP‐driven explainability, collectively enhancing reliability and clinical trust. Our model is superior to traditional tools like the Charlson Index and Framingham Score, with a higher XGBoost AUC range (Table 2). Most published heart disease mortality prediction models have an AUC between 0.7 and 0.85, and the performance of our model is comparable to recent cardiovascular risk prediction studies. Our multicohort validation (three independent cohorts) provides stronger evidence of generalizability. The NRI ranges from 0.190 to 0.335 compared to traditional scores, indicating significant improvement.

In summary, we constructed and externally validated an interpretable, generalizable ML model for predicting mortality in older heart disease patients across diverse populations. By incorporating geriatric‐focused predictors and explainable AI, the model identifies meaningful risks often missed by traditional tools. This approach supports more personalized, holistic cardiovascular risk assessment and may aid clinicians in improving care and outcomes for aging populations.

Several limitations should be acknowledged. Mortality outcomes were determined by follow‐up interviews rather than linkage to clinical death registries, potentially introducing classification errors. Despite careful harmonization, there may be residual measurement bias due to differences in survey instruments or cultural interpretations of health indicators, such as self‐rated health or cognitive test responses. Additionally, our study used observational data and is subject to unmeasured confounding. The model has not yet been validated prospectively in clinical workflows, and we did not evaluate cause‐specific mortality outcomes. None of the three cohorts provided data on specific causes of death. Only all‐cause mortality was recorded. Considering the high cardiovascular mortality rate among patients with heart disease (50%–70% reported in the literature), all‐cause mortality remained a valid endpoint. Future research should take into account competitive risk models. Finally, although SHAP facilitates interpretation, it does not establish causality, and further investigation is required to uncover mechanistic pathways. In the future, we will incorporate survival analysis methods, competitive risks, and apply deep learning methods to improve our model.

## 5. Conclusion

We developed a validated, interpretable ML model that predicts mortality in older heart disease patients across continents, highlighting the value of functional, cognitive, and self‐rated health measures for personalized risk assessment.

## Disclosure

All authors have confirmed their final authorship status.

## Conflicts of Interest

The authors declare no conflicts of interest.

## Funding

No funding was received for this manuscript.

## Supporting Information

Additional supporting information can be found online in the Supporting Information section.

## Supporting information


**Supporting Information 1** Figures S1–S4: Complete SHAP summary plots for all features. These supplementary figures illustrate the feature‐wise SHAP value distributions derived from the model: S1 (SHARE training set), S2 (SHARE testing set), S3 (HRS dataset), and S4 (CHARLS dataset). Each point indicates an individual instance, with color indicating the feature value. Features are ordered by their relative importance, with the highest‐ranking variables displayed at the top. Abbreviations: SHARE, the Survey of Health, Ageing and Retirement in Europe; HRS, the Health and Retirement Study; CHARLS, the China Health and Retirement Longitudinal Study; XGBoost, the extreme gradient boosting; SHAP, SHapley Additive exPlanations; BMI, body mass index.

**Supporting Information 2** Figures S5–S7: SHAP feature dependence plots for external validation datasets. These supplementary figures display feature dependence plots for the Top 9 important features in the external validation datasets: S5 (SHARE testing set), S6 (HRS dataset), and S7 (CHARLS dataset). These plots show how SHAP values (model impact) change with feature values. Abbreviations: SHARE, the Survey of Health, Ageing and Retirement in Europe; HRS, the Health and Retirement Study; CHARLS, the China Health and Retirement Longitudinal Study; SHAP, SHapley Additive exPlanations.

**Supporting Information 3** Figures S8–S10: SHAP waterfall plot comparisons for external validation datasets. These supplementary figures present comparisons of SHAP waterfall plots for representative high‐risk and low‐risk heart disease patients from external validation datasets: S8 (SHARE testing set), S9 (HRS dataset), and S10 (CHARLS dataset). Each plot shows how individual features contribute to pushing the prediction higher or lower than the base value. Abbreviations: SHARE, the Survey of Health, Ageing and Retirement in Europe; HRS, the Health and Retirement Study; CHARLS, the China Health and Retirement Longitudinal Study; XGBoost, the extreme gradient boosting; SHAP, SHapley Additive exPlanations; BMI, body mass index.

**Supporting Information 4** Table S1: Comprehensive comparison of all variables between different databases. This supplementary table provides a detailed comparison of all variables available across the CHARLS, SHARE, and HRS databases. Variables include demographic characteristics, physical measurements, chronic conditions, health assessments, cognitive function, lifestyle factors, functional status, and social participation. Data are presented as median [Q1, Q3] for continuous variables and *n* (percentage) for categorical variables. *p* values indicate statistical significance of differences between databases.

## Data Availability

The data that supports the findings of this study are available in the supporting information of this article.
