# Supplementary material for: A Multicohort Machine Learning Framework to Predict Mortality in Elderly Patients With Heart Disease: Insights From HARLS, SHARE, and HRS
Source: Cardiovasc Ther. 2026 Jan 2;2026:8040700. doi: 10.1155/cdr/8040700 (PMC12759112; doi:10.1155/cdr/8040700)
Supplement: Supplementary file 1 — Supporting Information 1 Figures S1–S4: Complete SHAP summary plots for all features. These supplementary figures illustrate the feature‐wise SHAP value distributions derived from the model: S1 (SHARE training set), S2 (SHARE testing set), S3 (HRS dataset), and S4 (CHARLS dataset). Each point indicates an individual instance, with color indicating the feature value. Features are ordered by their relative importance, with the highest‐ranking variables displayed at the top. Abbreviations: SHARE, the Survey of Health, Ageing and Retirement in Europe; HRS, the Health and Retirement Study; CHARLS, the China Health and Retirement Longitudinal Study; XGBoost, the extreme gradient boosting; SHAP, SHapley Additive exPlanations; BMI, body mass index. [file CDR-2026-8040700-s004.zip › Figure S1.pdf]

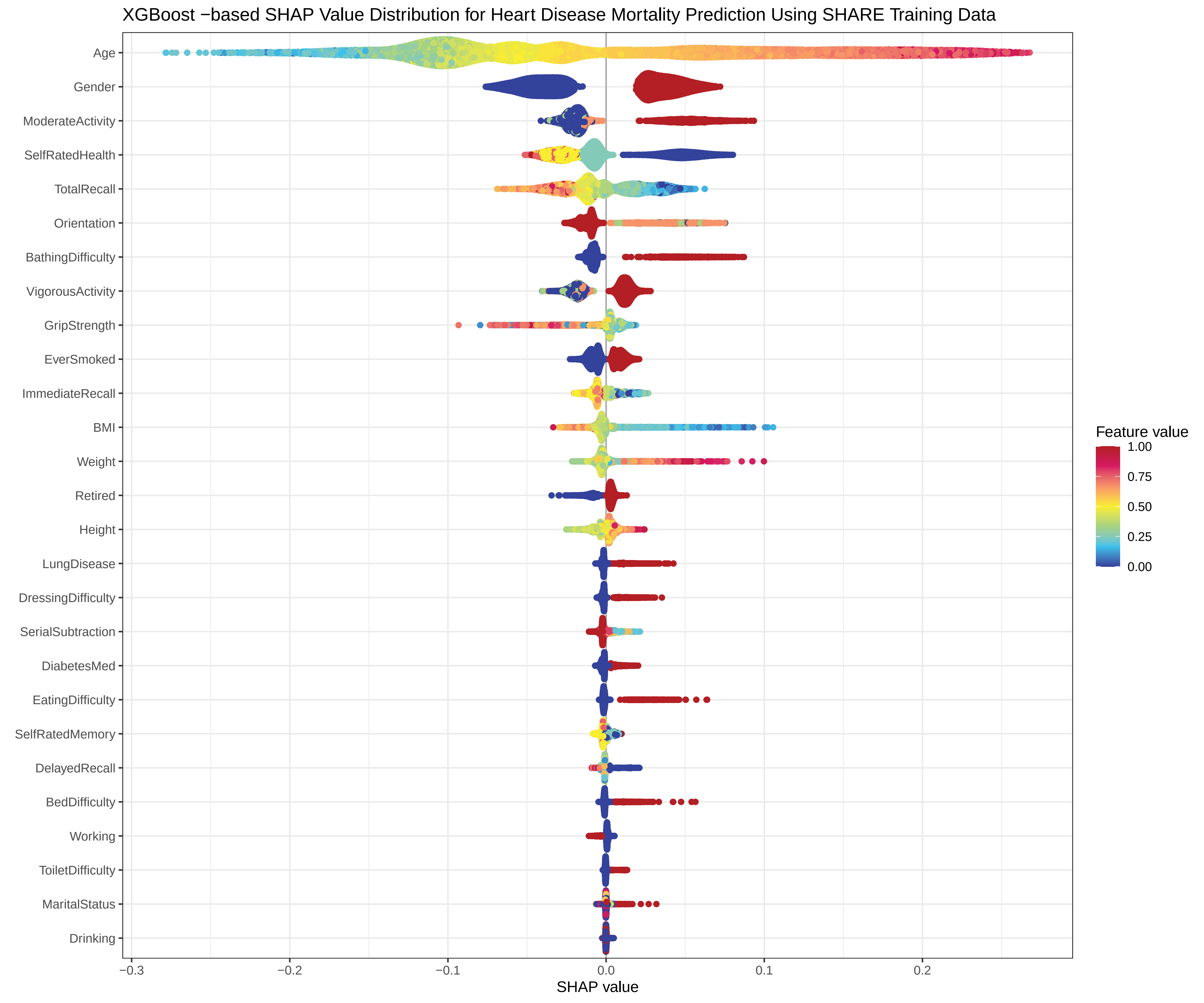
XGBoost −based SHAP Value Distribution for Heart Disease Mortality Prediction Using SHARE Training Data
Age
Gender
ModerateActivity
SelfRatedHealth
TotalRecall
Orientation
BathingDifficulty
VigorousActivity
GripStrength
EverSmoked
ImmediateRecall
BMI
Weight
Retired
Height
LungDisease
DressingDifficulty
SerialSubtraction
DiabetesMed
EatingDifficulty
SelfRatedMemory
DelayedRecall
BedDifficulty
Working
ToiletDifficulty
MaritalStatus
Drinking
−0.3
−0.2
−0.1
0.0
0.1
0.2
SHAP value
Feature value
1.00
0.75
0.50
0.25
0.00
