# Supplementary material for: A Multicohort Machine Learning Framework to Predict Mortality in Elderly Patients With Heart Disease: Insights From HARLS, SHARE, and HRS
Source: Cardiovasc Ther. 2026 Jan 2;2026:8040700. doi: 10.1155/cdr/8040700 (PMC12759112; doi:10.1155/cdr/8040700)

XGBoost −based SHAP Value Distribution for Heart Disease Mortality Prediction Using SHARE Testing Data

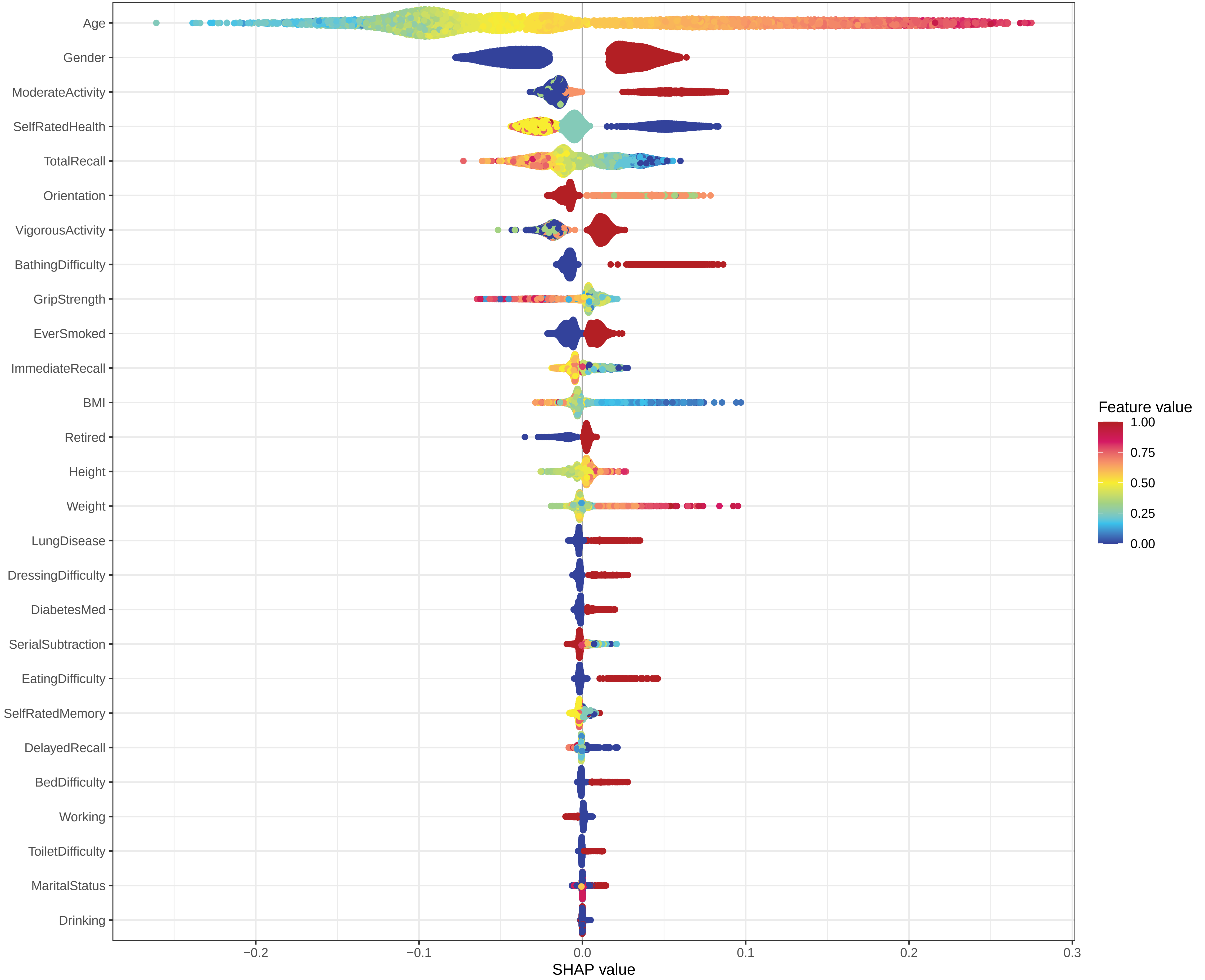

Supplement: Supplementary file 1 — Supporting Information 1 Figures S1–S4: Complete SHAP summary plots for all features. These supplementary figures illustrate the feature‐wise SHAP value distributions derived from the model: S1 (SHARE training set), S2 (SHARE testing set), S3 (HRS dataset), and S4 (CHARLS dataset). Each point indicates an individual instance, with color indicating the feature value. Features are ordered by their relative importance, with the highest‐ranking variables displayed at the top. Abbreviations: SHARE, the Survey of Health, Ageing and Retirement in Europe; HRS, the Health and Retirement Study; CHARLS, the China Health and Retirement Longitudinal Study; XGBoost, the extreme gradient boosting; SHAP, SHapley Additive exPlanations; BMI, body mass index. [file CDR-2026-8040700-s004.zip › Figure S2.pdf]
