# Supplementary material for: A Multicohort Machine Learning Framework to Predict Mortality in Elderly Patients With Heart Disease: Insights From HARLS, SHARE, and HRS
Source: Cardiovasc Ther. 2026 Jan 2;2026:8040700. doi: 10.1155/cdr/8040700 (PMC12759112; doi:10.1155/cdr/8040700)
Supplement: Supplementary file 2 — Supporting Information 2 Figures S5–S7: SHAP feature dependence plots for external validation datasets. These supplementary figures display feature dependence plots for the Top 9 important features in the external validation datasets: S5 (SHARE testing set), S6 (HRS dataset), and S7 (CHARLS dataset). These plots show how SHAP values (model impact) change with feature values. Abbreviations: SHARE, the Survey of Health, Ageing and Retirement in Europe; HRS, the Health and Retirement Study; CHARLS, the China Health and Retirement Longitudinal Study; SHAP, SHapley Additive exPlanations. [file CDR-2026-8040700-s001.zip › Figure S5.pdf]

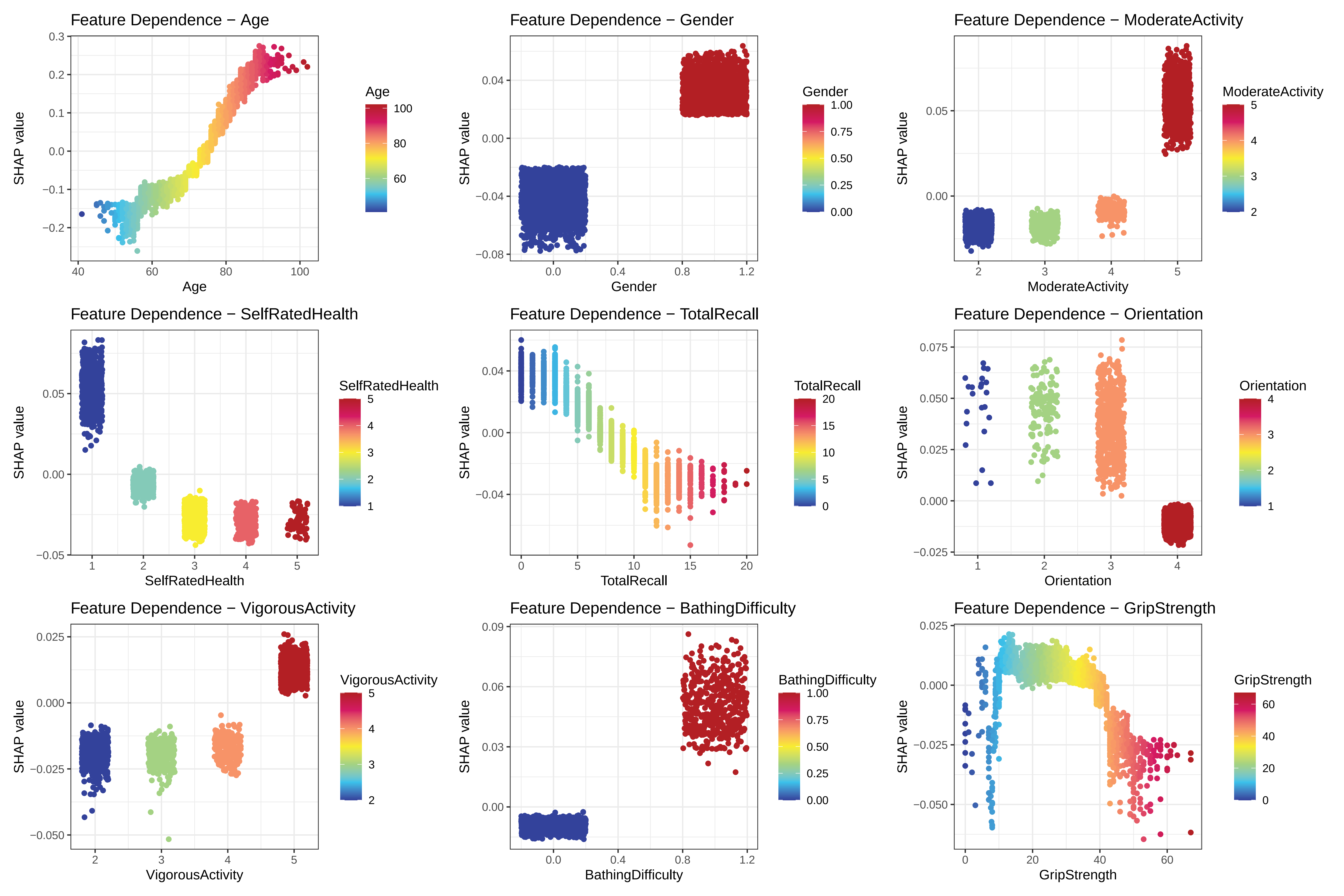

Feature Dependence − Age
SHAP value
0.3
0.2
0.1
0.0
−0.1
−0.2
40
60
80
100
Age
Age
100
80
60
Feature Dependence − Gender
SHAP value
0.04
0.00
−0.04
−0.08
0.0
0.4
0.8
1.2
Gender
Gender
1.00
0.75
0.50
0.25
0.00
Feature Dependence − ModerateActivity
SHAP value
0.05
0.00
2
3
4
5
ModerateActivity
ModerateActivity
5
4
3
2
Feature Dependence − SelfRatedHealth
SHAP value
0.05
0.00
−0.05
1
2
3
4
5
SelfRatedHealth
SelfRatedHealth
5
4
3
2
1
Feature Dependence − TotalRecall
SHAP value
0.04
0.00
−0.04
0
5
10
15
20
TotalRecall
TotalRecall
20
15
10
5
0
Feature Dependence − Orientation
SHAP value
0.075
0.050
0.025
0.000
−0.025
1
2
3
4
Orientation
Orientation
4
3
2
1
Feature Dependence − VigorousActivity
SHAP value
0.025
0.000
−0.025
−0.050
2
3
4
5
VigorousActivity
VigorousActivity
5
4
3
2
Feature Dependence − BathingDifficulty
SHAP value
0.09
0.06
0.03
0.00
0.0
0.4
0.8
1.2
BathingDifficulty
BathingDifficulty
1.00
0.75
0.50
0.25
0.00
Feature Dependence − GripStrength
SHAP value
0.025
0.000
−0.025
−0.050
0
20
40
60
GripStrength
GripStrength
60
40
20
0
