# Supplementary material for: A Multicohort Machine Learning Framework to Predict Mortality in Elderly Patients With Heart Disease: Insights From HARLS, SHARE, and HRS
Source: Cardiovasc Ther. 2026 Jan 2;2026:8040700. doi: 10.1155/cdr/8040700 (PMC12759112; doi:10.1155/cdr/8040700)
Supplement: Supplementary file 2 — Supporting Information 2 Figures S5–S7: SHAP feature dependence plots for external validation datasets. These supplementary figures display feature dependence plots for the Top 9 important features in the external validation datasets: S5 (SHARE testing set), S6 (HRS dataset), and S7 (CHARLS dataset). These plots show how SHAP values (model impact) change with feature values. Abbreviations: SHARE, the Survey of Health, Ageing and Retirement in Europe; HRS, the Health and Retirement Study; CHARLS, the China Health and Retirement Longitudinal Study; SHAP, SHapley Additive exPlanations. [file CDR-2026-8040700-s001.zip › Figure S7.pdf]

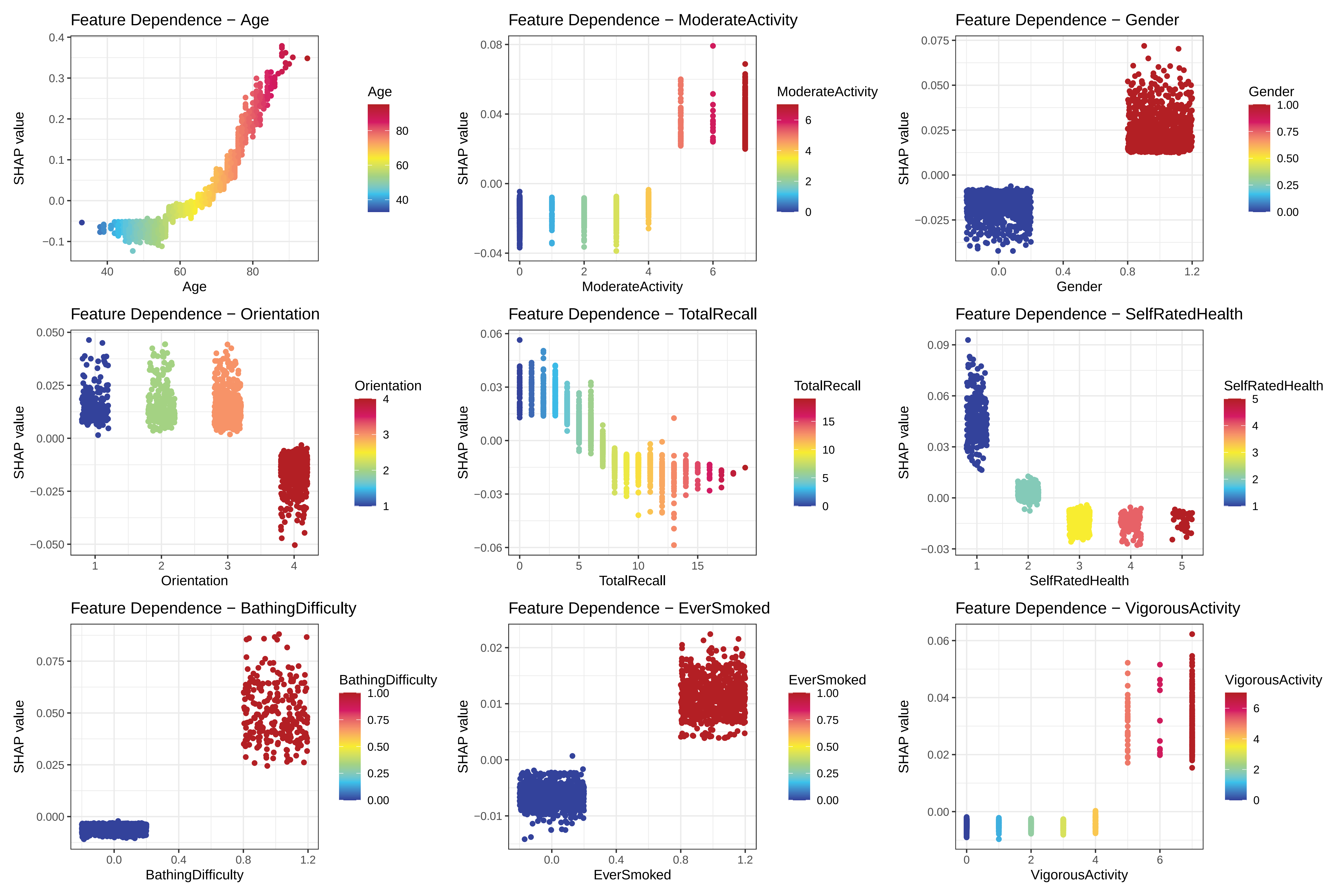

Feature Dependence − Age
Feature Dependence − ModerateActivity
Feature Dependence − Gender
Feature Dependence − Orientation
Feature Dependence − TotalRecall
Feature Dependence − SelfRatedHealth
Feature Dependence − BathingDifficulty
Feature Dependence − EverSmoked
Feature Dependence − VigorousActivity
SHAP value
Age
ModerateActivity
Gender
Orientation
TotalRecall
SelfRatedHealth
BathingDifficulty
EverSmoked
VigorousActivity
