# Supplementary material for: A Multicohort Machine Learning Framework to Predict Mortality in Elderly Patients With Heart Disease: Insights From HARLS, SHARE, and HRS
Source: Cardiovasc Ther. 2026 Jan 2;2026:8040700. doi: 10.1155/cdr/8040700 (PMC12759112; doi:10.1155/cdr/8040700)

XGBoost − SHAP Waterfall Plots for Heart Disease Mortality Prediction − share_test

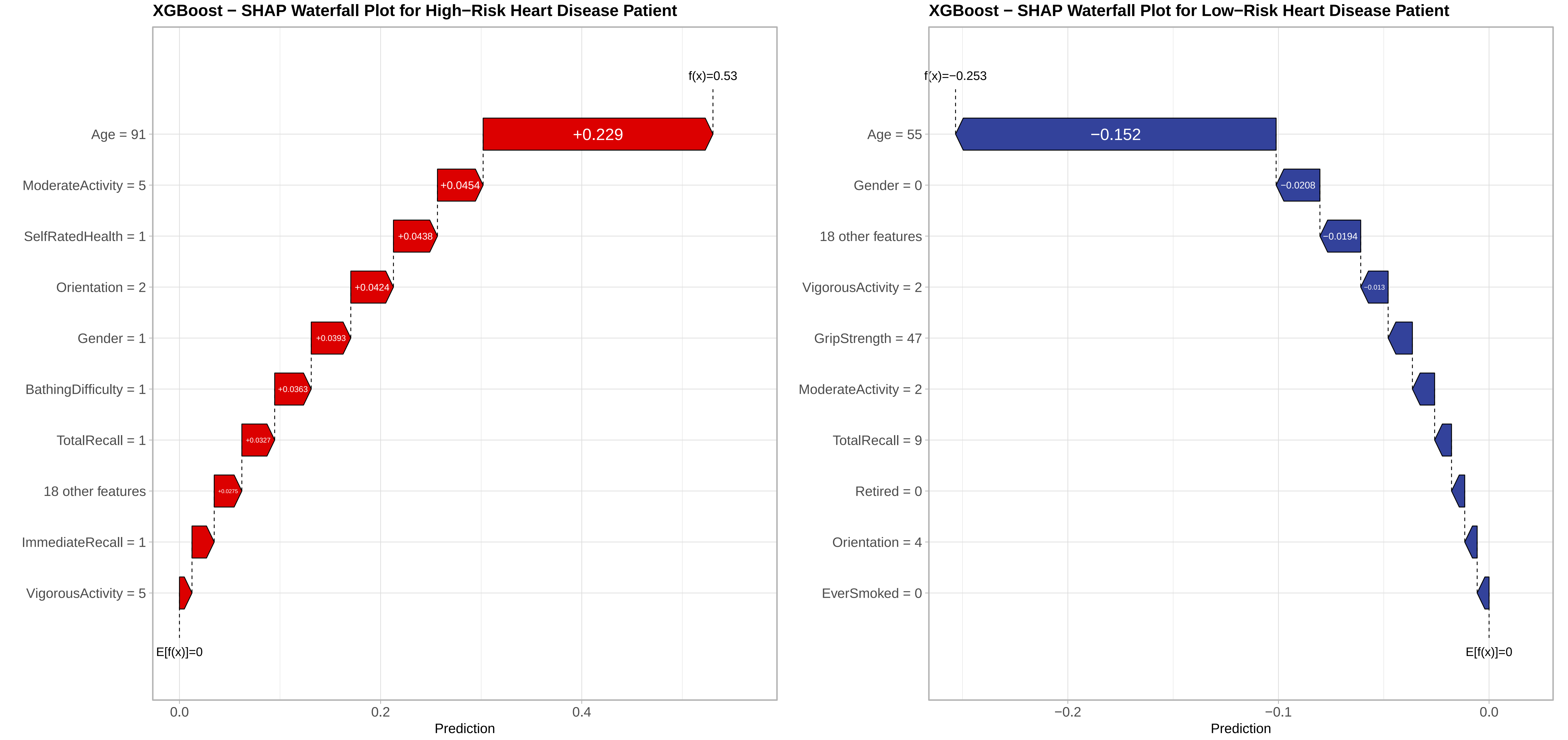

Supplement: Supplementary file 3 — Supporting Information 3 Figures S8–S10: SHAP waterfall plot comparisons for external validation datasets. These supplementary figures present comparisons of SHAP waterfall plots for representative high‐risk and low‐risk heart disease patients from external validation datasets: S8 (SHARE testing set), S9 (HRS dataset), and S10 (CHARLS dataset). Each plot shows how individual features contribute to pushing the prediction higher or lower than the base value. Abbreviations: SHARE, the Survey of Health, Ageing and Retirement in Europe; HRS, the Health and Retirement Study; CHARLS, the China Health and Retirement Longitudinal Study; XGBoost, the extreme gradient boosting; SHAP, SHapley Additive exPlanations; BMI, body mass index. [file CDR-2026-8040700-s003.zip › Figure S8.pdf]
