# Supplementary material for: A Multicohort Machine Learning Framework to Predict Mortality in Elderly Patients With Heart Disease: Insights From HARLS, SHARE, and HRS
Source: Cardiovasc Ther. 2026 Jan 2;2026:8040700. doi: 10.1155/cdr/8040700 (PMC12759112; doi:10.1155/cdr/8040700)
Supplement: Supplementary file 3 — Supporting Information 3 Figures S8–S10: SHAP waterfall plot comparisons for external validation datasets. These supplementary figures present comparisons of SHAP waterfall plots for representative high‐risk and low‐risk heart disease patients from external validation datasets: S8 (SHARE testing set), S9 (HRS dataset), and S10 (CHARLS dataset). Each plot shows how individual features contribute to pushing the prediction higher or lower than the base value. Abbreviations: SHARE, the Survey of Health, Ageing and Retirement in Europe; HRS, the Health and Retirement Study; CHARLS, the China Health and Retirement Longitudinal Study; XGBoost, the extreme gradient boosting; SHAP, SHapley Additive exPlanations; BMI, body mass index. [file CDR-2026-8040700-s003.zip › Figure S9.pdf]

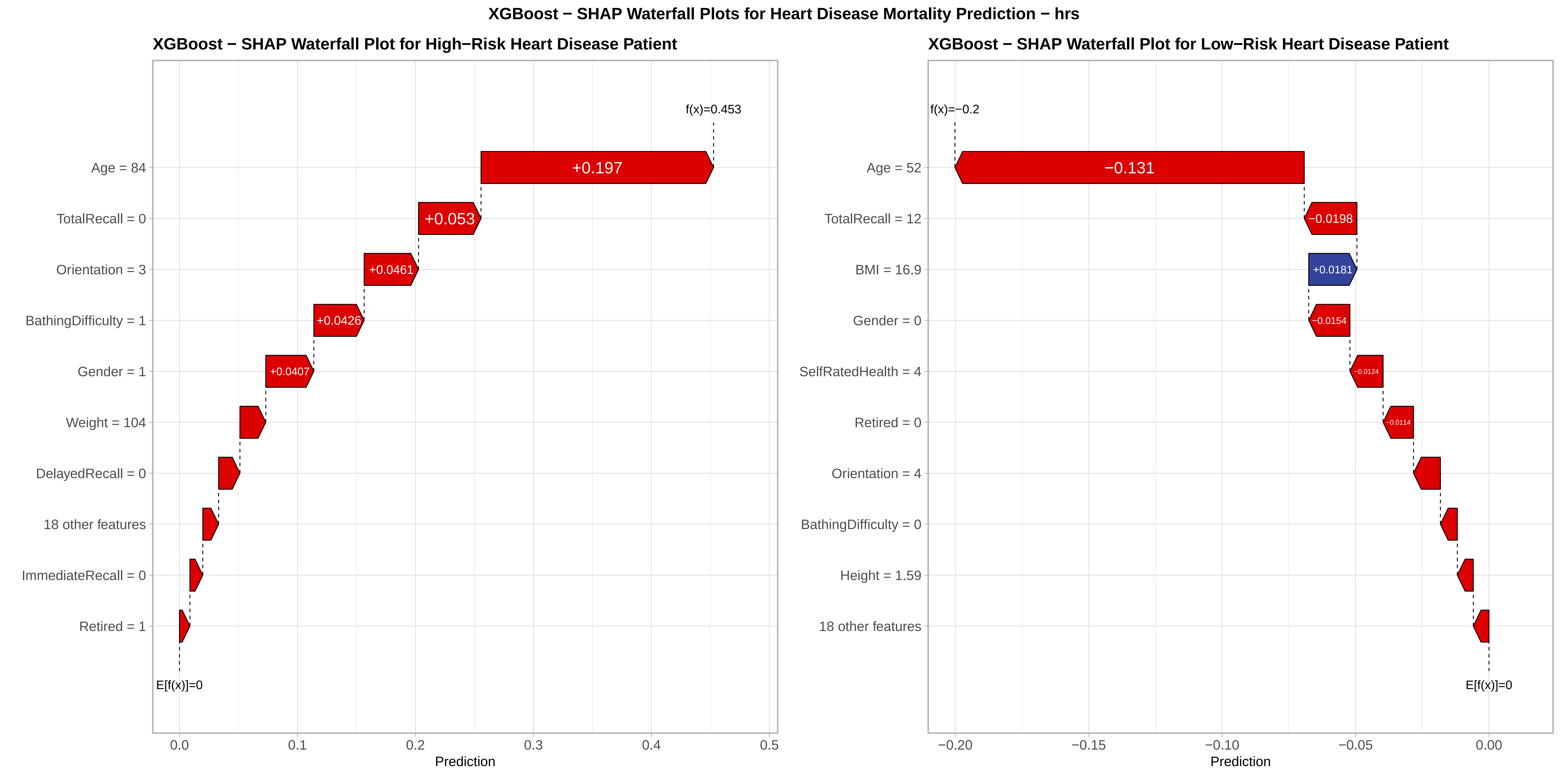
XGBoost − SHAP Waterfall Plots for Heart Disease Mortality Prediction − hrs
XGBoost − SHAP Waterfall Plot for High−Risk Heart Disease Patient
f(x)=0.453
Age = 84
+0.197
TotalRecall = 0
+0.053
Orientation = 3
+0.0461
BathingDifficulty = 1
+0.0426
Gender = 1
+0.0407
Weight = 104
DelayedRecall = 0
18 other features
ImmediateRecall = 0
Retired = 1
E[f(x)]=0
0.0
0.1
0.2
0.3
0.4
0.5
Prediction
XGBoost − SHAP Waterfall Plot for Low−Risk Heart Disease Patient
f(x)=−0.2
Age = 52
−0.131
TotalRecall = 12
−0.0198
BMI = 16.9
+0.0181
Gender = 0
−0.0154
SelfRatedHealth = 4
−0.0124
Retired = 0
−0.0114
Orientation = 4
BathingDifficulty = 0
Height = 1.59
18 other features
E[f(x)]=0
−0.20
−0.15
−0.10
−0.05
0.00
Prediction
