# Supplementary material for: A Multicohort Machine Learning Framework to Predict Mortality in Elderly Patients With Heart Disease: Insights From HARLS, SHARE, and HRS
Source: Cardiovasc Ther. 2026 Jan 2;2026:8040700. doi: 10.1155/cdr/8040700 (PMC12759112; doi:10.1155/cdr/8040700)
Supplement: Supplementary file 4 — Supporting Information 4 Table S1: Comprehensive comparison of all variables between different databases. This supplementary table provides a detailed comparison of all variables available across the CHARLS, SHARE, and HRS databases. Variables include demographic characteristics, physical measurements, chronic conditions, health assessments, cognitive function, lifestyle factors, functional status, and social participation. Data are presented as median [Q1, Q3] for continuous variables and n (percentage) for categorical variables. p values indicate statistical significance of differences between databases. [file CDR-2026-8040700-s002.docx]

# **Table S1. Comprehensive Comparison of All Variables Between Different Databases**

| **Characteristics** | **level** | **Overall (n=17893)** | **CHARLS (n= 2130)** | **HRS (n= 4835)** | **SHARE (n=10928)** | **P-value** | **SMD** |
| --- | --- | --- | --- | --- | --- | --- | --- |
| Age (years) (median [Q1,Q3]) |  | 72.00 [63.00, 79.00] | 61.00 [55.00, 69.00] | 73.00 [63.00, 80.00] | 73.00 [65.00, 80.00] | <0.001 | 0.679 |
| Gender | Female | 8964 ( 50.1) | 1269 ( 59.6) | 2470 ( 51.1) | 5225 ( 47.8) | <0.001 | 0.158 |
|  | Male | 8929 ( 49.9) | 861 ( 40.4) | 2365 ( 48.9) | 5703 ( 52.2) |  |  |
| Marital Status | Married | 11311 ( 63.2) | 1708 ( 80.2) | 2631 ( 54.4) | 6972 ( 63.8) | <0.001 | 0.552 |
|  | Married but separated | 180 ( 1.0) | 92 ( 4.3) | 87 ( 1.8) | 1 ( 0.0) |  |  |
|  | Cohabiting | 118 ( 0.7) | 0 ( 0.0) | 0 ( 0.0) | 118 ( 1.1) |  |  |
|  | Separated | 202 ( 1.1) | 6 ( 0.3) | 93 ( 1.9) | 103 ( 0.9) |  |  |
|  | Divorced | 1448 ( 8.1) | 23 ( 1.1) | 598 ( 12.4) | 827 ( 7.6) |  |  |
|  | Widowed | 3945 ( 22.0) | 283 ( 13.3) | 1239 ( 25.6) | 2423 ( 22.2) |  |  |
|  | Never married | 688 ( 3.8) | 18 ( 0.8) | 186 ( 3.8) | 484 ( 4.4) |  |  |
| Education Level | Primary education or below | 8333 ( 46.6) | 1837 ( 86.2) | 1116 ( 23.1) | 5380 ( 49.2) | <0.001 | 1.039 |
|  | Secondary education | 6734 ( 37.6) | 230 ( 10.8) | 2867 ( 59.3) | 3637 ( 33.3) |  |  |
|  | Tertiary education | 2826 ( 15.8) | 63 ( 3.0) | 852 ( 17.6) | 1911 ( 17.5) |  |  |
| Height (m) (median [Q1,Q3]) |  | 1.66 [1.59, 1.73] | 1.58 [1.52, 1.65] | 1.66 [1.58, 1.73] | 1.68 [1.61, 1.75] | <0.001 | 0.678 |
| Weight (kg) (median [Q1,Q3]) |  | 75.00 [65.00, 86.86] | 62.00 [54.00, 70.10] | 77.56 [66.41, 91.22] | 77.00 [68.00, 87.00] | <0.001 | 0.785 |
| BMI (kg/m²) (median [Q1,Q3]) |  | 27.00 [24.24, 30.46] | 24.50 [22.00, 27.25] | 28.15 [25.17, 32.42] | 27.06 [24.44, 30.39] | <0.001 | 0.606 |
| Grip Strength (kg) (median [Q1,Q3]) |  | 26.50 [19.30, 35.90] | 28.50 [22.00, 36.00] | 27.00 [20.00, 35.50] | 26.00 [18.78, 36.00] | <0.001 | 0.097 |
| Stroke | No | 15172 ( 84.8) | 2015 ( 94.6) | 3939 ( 81.5) | 9218 ( 84.4) | <0.001 | 0.276 |
|  | Yes | 2721 ( 15.2) | 115 ( 5.4) | 896 ( 18.5) | 1710 ( 15.6) |  |  |
| Hypertension | No | 5843 ( 32.7) | 1017 ( 47.7) | 1179 ( 24.4) | 3647 ( 33.4) | <0.001 | 0.332 |
|  | Yes | 12050 ( 67.3) | 1113 ( 52.3) | 3656 ( 75.6) | 7281 ( 66.6) |  |  |
| Diabetes | No | 13156 ( 73.5) | 1837 ( 86.2) | 3250 ( 67.2) | 8069 ( 73.8) | <0.001 | 0.307 |
|  | Yes | 4737 ( 26.5) | 293 ( 13.8) | 1585 ( 32.8) | 2859 ( 26.2) |  |  |
| Cancer | No | 15512 ( 86.7) | 2099 ( 98.5) | 3895 ( 80.6) | 9518 ( 87.1) | <0.001 | 0.416 |
|  | Yes | 2381 ( 13.3) | 31 ( 1.5) | 940 ( 19.4) | 1410 ( 12.9) |  |  |
| Lung Disease | No | 14832 ( 82.9) | 1760 ( 82.6) | 4008 ( 82.9) | 9064 ( 82.9) | 0.940 | 0.006 |
|  | Yes | 3061 ( 17.1) | 370 ( 17.4) | 827 ( 17.1) | 1864 ( 17.1) |  |  |
| Arthritis | No | 8608 ( 48.1) | 1136 ( 53.3) | 1426 ( 29.5) | 6046 ( 55.3) | <0.001 | 0.360 |
|  | Yes | 9285 ( 51.9) | 994 ( 46.7) | 3409 ( 70.5) | 4882 ( 44.7) |  |  |
| Self-rated Health | Very poor | 3841 ( 21.5) | 224 ( 10.5) | 814 ( 16.8) | 2803 ( 25.6) | <0.001 | 0.448 |
|  | Poor | 6659 ( 37.2) | 842 ( 39.5) | 1445 ( 29.9) | 4372 ( 40.0) |  |  |
|  | Average | 5342 ( 29.9) | 887 ( 41.6) | 1583 ( 32.7) | 2872 ( 26.3) |  |  |
|  | Good | 1654 ( 9.2) | 137 ( 6.4) | 842 ( 17.4) | 675 ( 6.2) |  |  |
|  | Excellent | 397 ( 2.2) | 40 ( 1.9) | 151 ( 3.1) | 206 ( 1.9) |  |  |
| Depression | No | 11586 ( 64.8) | 1088 ( 51.1) | 3806 ( 78.7) | 6692 ( 61.2) | <0.001 | 0.400 |
|  | Yes | 6307 ( 35.2) | 1042 ( 48.9) | 1029 ( 21.3) | 4236 ( 38.8) |  |  |
| Self-rated Memory | Very poor | 2695 ( 15.1) | 914 ( 42.9) | 448 ( 9.3) | 1333 ( 12.2) | <0.001 | 0.778 |
|  | Poor | 5893 ( 32.9) | 881 ( 41.4) | 1466 ( 30.3) | 3546 ( 32.4) |  |  |
|  | Average | 6464 ( 36.1) | 257 ( 12.1) | 1891 ( 39.1) | 4316 ( 39.5) |  |  |
|  | Good | 2227 ( 12.4) | 72 ( 3.4) | 826 ( 17.1) | 1329 ( 12.2) |  |  |
|  | Excellent | 614 ( 3.4) | 6 ( 0.3) | 204 ( 4.2) | 404 ( 3.7) |  |  |
| Immediate Recall Score (median [Q1,Q3]) |  | 5.00 [3.00, 6.00] | 4.00 [3.00, 5.00] | 5.00 [4.00, 6.00] | 5.00 [3.00, 6.00] | <0.001 | 0.330 |
| Delayed Recall Score (median [Q1,Q3]) |  | 3.00 [2.00, 5.00] | 3.00 [2.00, 4.00] | 4.00 [3.00, 5.00] | 3.00 [2.00, 5.00] | <0.001 | 0.257 |
| Total Recall Score (median [Q1,Q3]) |  | 8.00 [5.00, 10.00] | 7.00 [5.00, 9.00] | 9.00 [6.00, 11.00] | 8.00 [5.00, 10.00] | <0.001 | 0.312 |
| Serial Subtraction Score (median [Q1,Q3]) |  | 4.00 [3.00, 5.00] | 4.00 [2.00, 5.00] | 4.00 [2.00, 5.00] | 5.00 [3.00, 5.00] | <0.001 | 0.312 |
| Orientation Score (median [Q1,Q3]) |  | 4.00 [3.00, 4.00] | 3.00 [2.00, 4.00] | 4.00 [3.00, 4.00] | 4.00 [4.00, 4.00] | <0.001 | 0.516 |
| Ever Smoked | No | 8868 ( 49.6) | 1353 ( 63.5) | 1832 ( 37.9) | 5683 ( 52.0) | <0.001 | 0.351 |
|  | Yes | 9025 ( 50.4) | 777 ( 36.5) | 3003 ( 62.1) | 5245 ( 48.0) |  |  |
| Current Smoker | No | 15322 ( 85.6) | 1635 ( 76.8) | 4231 ( 87.5) | 9456 ( 86.5) | <0.001 | 0.189 |
|  | Yes | 2571 ( 14.4) | 495 ( 23.2) | 604 ( 12.5) | 1472 ( 13.5) |  |  |
| Alcohol Consumption | No | 11139 ( 62.3) | 1637 ( 76.9) | 2608 ( 53.9) | 6894 ( 63.1) | <0.001 | 0.329 |
|  | Yes | 6754 ( 37.7) | 493 ( 23.1) | 2227 ( 46.1) | 4034 ( 36.9) |  |  |
| Vigorous Activity Frequency (median [Q1,Q3]) |  | 3.00 [0.00, 5.00] | 0.00 [0.00, 2.00] | 0.00 [0.00, 1.00] | 5.00 [3.00, 5.00] | <0.001 | 1.510 |
| Moderate Activity Frequency (median [Q1,Q3]) |  | 2.00 [2.00, 3.00] | 1.00 [0.00, 7.00] | 2.00 [0.00, 3.00] | 2.00 [2.00, 4.00] | <0.001 | 0.545 |
| Dressing Difficulty | No | 14865 ( 83.1) | 1943 ( 91.2) | 3856 ( 79.8) | 9066 ( 83.0) | <0.001 | 0.220 |
|  | Yes | 3028 ( 16.9) | 187 ( 8.8) | 979 ( 20.2) | 1862 ( 17.0) |  |  |
| Bathing Difficulty | No | 15199 ( 84.9) | 1878 ( 88.2) | 4040 ( 83.6) | 9281 ( 84.9) | <0.001 | 0.088 |
|  | Yes | 2694 ( 15.1) | 252 ( 11.8) | 795 ( 16.4) | 1647 ( 15.1) |  |  |
| Bed Difficulty | No | 16042 ( 89.7) | 1939 ( 91.0) | 4190 ( 86.7) | 9913 ( 90.7) | <0.001 | 0.093 |
|  | Yes | 1851 ( 10.3) | 191 ( 9.0) | 645 ( 13.3) | 1015 ( 9.3) |  |  |
| Toilet Difficulty | No | 16135 ( 90.2) | 1718 ( 80.7) | 4197 ( 86.8) | 10220 ( 93.5) | <0.001 | 0.262 |
|  | Yes | 1758 ( 9.8) | 412 ( 19.3) | 638 ( 13.2) | 708 ( 6.5) |  |  |
| Working | No | 14259 ( 79.7) | 1207 ( 56.7) | 3782 ( 78.2) | 9270 ( 84.8) | <0.001 | 0.432 |
|  | Yes | 3634 ( 20.3) | 923 ( 43.3) | 1053 ( 21.8) | 1658 ( 15.2) |  |  |
| Retired | No | 6243 ( 35.8) | 1611 ( 75.6) | 1948 ( 44.3) | 2684 ( 24.6) | <0.001 | 0.763 |
|  | Yes | 11214 ( 64.2) | 519 ( 24.4) | 2451 ( 55.7) | 8244 ( 75.4) |  |  |
| Social Participation | No | 11148 ( 62.3) | 1114 ( 52.3) | 2865 ( 59.3) | 7169 ( 65.6) | <0.001 | 0.182 |
|  | Yes | 6745 ( 37.7) | 1016 ( 47.7) | 1970 ( 40.7) | 3759 ( 34.4) |  |  |
| Hypertension Medication | No | 6577 ( 36.8) | 1268 ( 59.5) | 1320 ( 27.3) | 3989 ( 36.5) | <0.001 | 0.453 |
|  | Yes | 11316 ( 63.2) | 862 ( 40.5) | 3515 ( 72.7) | 6939 ( 63.5) |  |  |
| Diabetes Medication | No | 14073 ( 78.7) | 1945 ( 91.3) | 3463 ( 71.6) | 8665 ( 79.3) | <0.001 | 0.349 |
|  | Yes | 3820 ( 21.3) | 185 ( 8.7) | 1372 ( 28.4) | 2263 ( 20.7) |  |  |
| Survival Status | No | 11863 ( 66.3) | 1711 ( 80.3) | 2407 ( 49.8) | 7745 ( 70.9) | <0.001 | 0.446 |
|  | Yes | 6030 ( 33.7) | 419 ( 19.7) | 2428 ( 50.2) | 3183 ( 29.1) |  |  |

Abbreviations: CHARLS, the China Health and Retirement Longitudinal Study; HRS, The Health and Retirement Study; SHARE, the Survey of Health, Ageing and Retirement in Europe; SMD, Standard Mean Difference.
